# TapA acts as specific chaperone in TasA filament formation by strand complementation

**DOI:** 10.1073/pnas.2217070120

**Published:** 2023-04-17

**Authors:** Yvette Roske, Florian Lindemann, Anne Diehl, Nils Cremer, Victoria A. Higman, Brigitte Schlegel, Martina Leidert, Kristina Driller, Kürşad Turgay, Peter Schmieder, Udo Heinemann, Hartmut Oschkinat

**Affiliations:** ^a^Structural Biology, Max Delbrück Center for Molecular Medicine, 13125 Berlin, Germany; ^b^Leibniz-Forschungsinstitut für Molekulare Pharmakologie, 13125 Berlin, Germany; ^c^Department for Molecular and Cell Biology, Leicester Institute of Structural and Chemical Biology, University of Leicester, Leicester LE1 7HB, United Kingdom; ^d^Max Planck Unit for the Science of Pathogens, 10117 Berlin, Germany; ^e^Institute of Microbiology, Leibniz Universität Hannover, 30419 Hannover, Germany; ^f^Institut für Chemie und Biochemie, Freie Universität Berlin, 14195 Berlin, Germany

**Keywords:** *Bacillus subtilis*, biofilm, TasA, TapA/YqxM, structure

## Abstract

Studying mechanisms of bacterial biofilm generation is of vital importance to understanding bacterial cell–cell communication, multicellular cohabitation principles, and the higher resilience of microorganisms in a biofilm against antibiotics. Biofilms of the nonpathogenic, gram-positive soil bacterium *Bacillus subtilis* serve as a model system with biotechnological potential toward plant protection. Its major extracellular matrix protein components are TasA and TapA. The nature of TasA filaments has been of debate, and several forms, amyloidic and non-Thioflavin T-stainable have been observed. Here, we present the three-dimensional structure of TapA and uncover the mechanism of TapA-supported growth of nonamyloidic TasA filaments. By analytical ultracentrifugation and NMR, we demonstrate TapA-dependent acceleration of filament formation from solutions of folded TasA. Solid-state NMR revealed intercalation of the N-terminal TasA peptide segment into subsequent protomers to form a filament composed of β-sandwich subunits. The secondary structure around the intercalated N-terminal strand β0 is conserved between filamentous TasA and the Fim and Pap proteins, which form bacterial type I pili, demonstrating such construction principles in a gram-positive organism. Analogous to the chaperones of the chaperone-usher pathway, the role of TapA is in donating its N terminus to serve for TasA folding into an Ig domain-similar filament structure by donor-strand complementation. According to NMR and since the V-set Ig fold of TapA is already complete, its participation within a filament beyond initiation is unlikely. Intriguingly, the most conserved residues in TasA-like proteins (camelysines) of *Bacillaceae* are located within the protomer interface.

## Significance

Bacteria on surfaces often protect themselves by forming biofilms, with huge negative impact on health and the economy. Biofilms are stabilized by protein-based super structures in the extracellular matrix and means to suppress or resolve them are urgently needed given the looming health-crisis due to antibiotic-resistant bacterial strains. Our model organism *Bacillus subtilis* has disease-causing relatives such *Bacillus cereus* and *Bacillus anthracis*, and thus provides a means to investigate common biofilm formation mechanisms. It produces two biofilm proteins, TasA (major component) and TapA (minor component). We have analyzed TapA-triggered filament formation by TasA and find that protomers connect via the donor-strand complementation mechanism of gram-negative bacteria. These results form the basis for the development of effective strategies against biofilm formation.

Most microorganisms form sessile multicellular biofilms in which they are protected against stress by a gel-like matrix composed of proteinaceous fibrils, various extracellular polysaccharides and often DNA (1). *Bacillus subtilis* expresses TasA (translocation-dependent antimicrobial spore component) as the major biofilm protein (2), which aided by TapA (TasA anchoring/assembly protein, YqxM) (3) can convert into stable oligomeric structures to form the biofilm matrix (4, 5). Whereas biofilms often contain β-helix type protofibrils that can be stained by Thioflavin T (ThT), e.g., curli of *Escherichia coli* (6), TasA forms different architectures in vitro depending on experimental conditions (2, 7–9). ThT-stainable fibrils were initially generated from acid-treated protein at low pH (2) and recently also from unfolded protein at neutral pH (10), whereas TasA from natural sources was found early on to form stable, nonamyloid oligomers (7, 9).

The importance of TapA (referring to the mature protein, residues 44 to 253, if not otherwise indicated) for robust biofilm formation was already recognized when TasA (mature protein, residues 28 to 261) was identified as the major biofilm protein of *B. subtilis* (2, 11, 12). A role as polymerization nucleator was proposed for TapA (3, 13), whereby its N terminus (14, 15) is considered essential. Outside the cell, TapA is found to colocalize with the membrane (13), and individual observations also identified it in fibrils (13). TapA is present at a much lower abundance than TasA, similar to the signal peptidase SipW (13). The coexpression of TapA and SipW and the overlap of their stop and start codons, respectively, hints at a functional and structural association (16). TapA was characterized as a two-domain protein with an unstructured N terminus (17).

Recently, a structural basis for the different TasA forms became available. A three-dimensional structure of monomeric TasA_G28-239_ was obtained by X-ray crystallography (8). ThT-stainable fibrils were investigated by solid-state NMR (10), and in parallel to this study nonamyloid filaments by cryo-EM (18) were studied at a resolution of 3.5 Å. Furthermore, a structural relationship between archaeal and bacterial biofilms was suggested on the basis of a 4.0 Å cryo-EM structure of *Pyrobaculum calidifontis* bundling pili (19).

TasA homologs (called camelysins, *SI Appendix*, Fig. S1) occur in various branches of bacteria and archaea (19) but TapA is not even conserved within the *Bacillus* genus. On the other hand, *Bacillus cereus* has two camelysins, *calY1* and *calY2*, encoded in one operon together with the specific signal peptidase SipW *(sipW-calY1-bc_1280-calY2*) instead of the *tapA*-*sipW*-*tasA* combination found in *B. subtilis* (*SI Appendix*, Fig. S1, entries for *B. cereus* in lines 2 and 3). It is likely that one of the two camelysins in *B. cereus* adopts the role as TapA in *B. subtilis* (3). Since bacteria take advantage of mixed biofilms, sometimes even involving fungi (4, 20), conservation of TasA-like proteins is highly informative. *B. cereus*, for example, induces expression of *B. subtilis* biofilm genes via the secretion of thiocillins (21). Cross-kingdom biofilm formation of *B. subtilis* involving hyphae of fungi such as *Aspergillus niger* and *Agaricus bisporus* has also been linked to TasA (22). In this context, it could be useful to identify indicators for joint biofilm formation.

Our aim is to understand the role of TapA in *B. subtilis* biofilm formation and to derive a mechanism for TapA-dependent TasA filament formation. Interactions of TapA with folded TasA are initially monitored by analytical ultracentrifugation (AUC) in a quantitative manner, supplemented by solution NMR data. To provide insight from a structural point, we determined the X-ray structure of a TapA core domain construct and the secondary structure of TasA filaments by solid-state NMR, based on near-complete backbone resonance assignments. Crucial intermolecular distances within the filament were monitored by mixing two TasA proteins with different labelling patterns. Based on our biophysical and structural results, a mechanism of TapA-initiated TasA filament formation is presented. For the gram-positive bacteria system, we demonstrate that TapA initiates TasA oligomerization similar to the chaperones of the chaperone-usher pathway of pilus assembly in gram-negative bacteria.

## Results

### TapA Enhances TasA Oligomerization.

Solutions of monomeric TasA are known to be stable at neutral pH for several days or weeks, but may form non-ThT-stainable filaments when concentrated over a membrane composed of regenerated cellulose (8, 9). By contrast, stainable fibrils, presumably amyloid-like, are formed at low pH from unfolded protein as shown by Romero et al. (2). TapA is known to induce or accelerate the formation of native TasA filaments (15) or fibrils (14). Here, we characterize this process on a molecular level for mature TapA and individual sections of its sequence, mainly using AUC and NMR, to understand whether TapA is merely a catalyst or becomes part of TasA filaments as proposed earlier (3, 10).

**Fig. 1.**
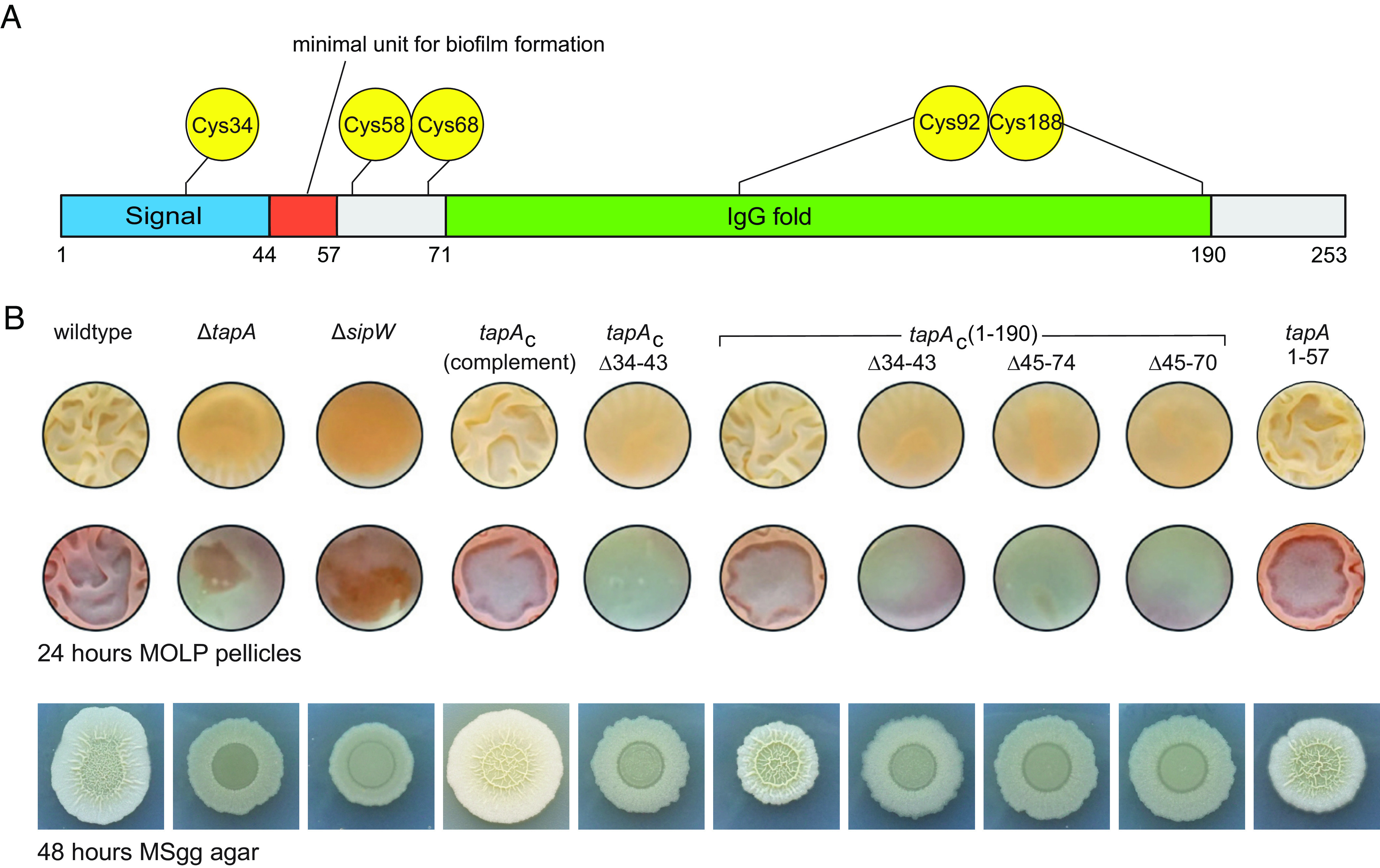
Structure of TapA and the impact of N- and C-terminal deletions on biofilm formation. (*A*) Schematic domain composition of TapA. The signal sequence (AA 1-43) is shown in blue, the minimal unit (AA 44-57) for biofilm formation (15) in red, the folded domain in green, and the positions of the five cysteines C34, C58, C68, C92, and C188 are indicated. (*B*) Biofilm formation of TapA-modified *B. subtilis* strains grown on MOLP medium as pellicles (two top rows, the second row includes staining by Coomassie blue and Congo red) or on MSgg medium agar plates (bottom row). The strain modifications are indicated, see text for further explanations.

Functional units of the TapA sequence were analyzed by employing N- and C-terminal truncations. Secondary structure prediction by Jpred4 (23) and later AlphaFold (24) both indicated a folded domain between residues K72 and D189, including a disulfide bond (Cys92-Cys188) (Fig. 1*A*), and a large unstructured region beyond residue E190. The N terminus was considered flexible. Furthermore, different starting points of the mature protein [A33 (10, 17, 25) or A44 (15)] are reported in the literature, with a large number of experiments performed on a TapA construct starting with A33, so that we also aimed to investigate more precisely the signal peptide cleavage site leading to mature TapA. The effects of the sequence alterations were examined by growing biofilms on MOLP medium as pellicles or on MSgg medium-containing agar plates (for the wild-type see Fig. 1*B*, column 1). Deletion of *tapA* or *sipW* both corrupt the biofilms (see Fig. 1*B*, columns 2 and 3). The use of *B. subtilis ∆tasA* operon strains complemented in *trans* with intact *tapA* in the *lacA* chromosomal locus and the *sipW*-*tasA* genes in the *amyE* locus (*tapA* complement, Fig. 1*B*, column 4) restores the wild-type biofilm phenotype, as well as the truncated *tap*A_1-190_ (Fig. 1*B*, column 6). Deleting amino acid residues 34 to 43 in both wild-type and *tap*A_1-190_ (*tapA_C_* Δ34-43 and *tapA*_1-190_ Δ34-43, Fig. 1*B*, columns 5 and 7) shows no biofilm formation, since signal peptide recognition, processing, and secretion could be affected by this deletion, indicating that the mature protein starts at A44. The deletions of amino acids 45 to 74 and 45 to 70 from the truncated protein TapA_1-190_ (Fig. 1*B*, columns 8 and 9) also impairs the biofilm. Furthermore, a strain secreting only residues 44 to 57 of the N terminus after cleavage of the signal peptide (*tapA*_1-57_, Fig. 1*B* column 10) showed proper biofilm formation as observed previously (14, 15). These experiments confirm A44 as the N terminus of mature TapA, and the crucial importance of N-terminal residues for biofilm formation. Deleting all residues beyond E190 did not change the appearance of the biofilms compared to wildtype TapA.

To determine conditions for in vitro TapA–TasA interaction experiments, the dependency on pH and/or salt concentration was investigated by isothermal titration calorimetry (ITC), interpreting the occurring heat changes solely as indicators of folding events combined with oligomerization. At pH 3 and 50 mM NaCl heat development was observed for the titration of TapA into TasA (*SI Appendix*, Fig. S2*A*), whereas the titration at pH 7 and 50 mM NaCl did not yield a response (*SI Appendix*, Fig. S2*B*). Increasing the salinity to 150 mM NaCl at pH 7.0 resulted again in a heat change (*SI Appendix*, Fig. S2*C*), demonstrating a considerable influence of salt concentration on TapA-dependent TasA oligomerization at neutral pH.

To ensure purity of the samples investigated below, the nature of the occurring TasA oligomers, potentially consisting of fibrils (ThT-stainable) and/or filaments (non-ThT-stainable) (15) were investigated in ThT-staining experiments (*SI Appendix*, Fig. S3) under very similar conditions. At pH 7, 20 °C, and 150 mM salt (buffer alone in first column), TasA forms nonstainable filaments (second column). The mixture of TapA_44-190_ and TasA as well as the functional construct TapA_44-190_ alone do not show relevant ThT responses either (columns 3 and 4, respectively). At low salt (50 mM), 40 °C, and neutral pH, small amounts of TasA progress toward stainable oligomeric states within 24 h (column 5). At low pH, intense fibril formation takes place within the first four hours (columns 6 and 7). TapA alone does not convert to stainable fibrils at low pH (column 8) and the C-terminally truncated variants of TapA show very similar effects to the full-length, mature protein (columns 9 and 10). In summary, fibrillar states of TasA are not observed under the conditions applied in our experiments at neutral pH but non-ThT-stainable filaments are formed.

**Fig. 2.**
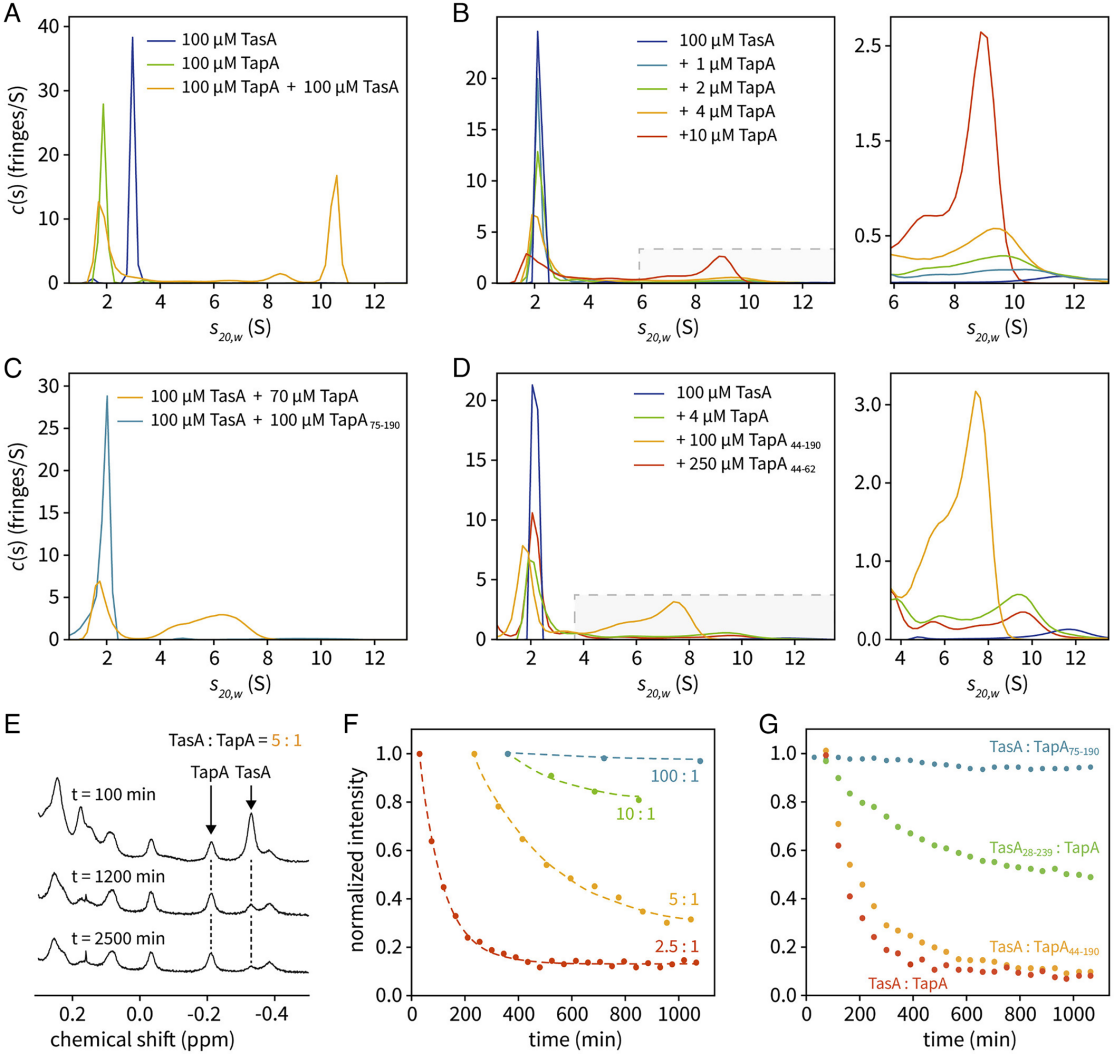
Interaction of TapA and TasA at 20 °C, pH 7, 150 mM NaCl. (*A*–*D*) AUC experiments performed with TasA_28-261_ and TapA_44-253_, unless otherwise noted. (*A*) TapA and ^2^H, ^13^C, ^15^N-labeled TasA alone (green and blue peaks, respectively), and of a mixture of both consisting of 100 µM TapA and 100 µM TasA (orange). (*B*) 100 µM TasA was analyzed separately or in combination with 1, 2, 4, and 10 µM TapA. The gray-shaded area is shown enlarged to the right. (*C*) AUC of 100 µM TasA with 100 µM TapA_75-190_ (turquoise) and of 100 µM TasA with 70 µM TapA for comparison (orange). (*D*) Evaluation of the effect of 250 µM C58SF61A-TapA_44-62_ on 100 µM TasA (brown curve, see also enlargement to the right). Oligomer formation corresponds to less than 4 µM full-length TapA (green). The variant with deleted C terminus, TapA_44-190_, shows wildtype behavior (orange). (*E*) ^1^H NMR spectra of methyl group region from a 5:1 mixture of TasA:TapA, with TasA at 125 µM. The intensities of TapA and TasA signals around −0.2 ppm and −0.35 ppm, respectively, were followed over time. While the signal of TapA remains nearly unaltered, the signal of TasA is decaying. (*F*) Time course of the disappearance of folded, monomeric TasA at different TasA:TapA ratios, with TasA at 125 µM, obtained on the spectra shown in (*E*). Signals are normalized to the first spectrum, time points refer to the time of mixing the solutions. (*G*) Time course of the disappearance of folded, monomeric TasA at a TasA:TapA ratio of 5:1, with TasA at 250 µM. TapA_75-190_ did not have any effect, for further information see text.

TapA-mediated oligomerization of folded, monomeric TasA was then analyzed by sedimentation velocity AUC experiments at 20 °C, 150 mM NaCl, and pH 7 (*SI Appendix*, Table S1) with runs lasting 17 h each. TapA and TasA were first investigated separately, yielding the expected sedimentation coefficient distributions of monomers, with single peaks at an S_20,w_ value of around 1.9 for TapA, and around 3.0 for ^2^H, ^13^C, ^15^N-labeled TasA (Fig. 2*A*) (with labeling, TasA occurs at a larger *s*-value; otherwise it would have occurred at around 2.1 resulting in overlap), and no oligomers were observed. A 1:1 mixture of both, however, revealed the presence of oligomers (Fig. 2*A*, orange curve) and the peak of monomeric TasA became vanishingly small. The TapA peak area was reduced to 84% +/− 1% as indicated by the comparison of the integrals of its green and orange monomer peaks, revealing a participation of TapA in oligomeric states. The three experiments shown in Fig. 2*A* were performed in the same run in parallel under the same conditions. The gray columns in *SI Appendix*, Fig. S3 are obtained after AUC and shaking up the samples. They indicate that amyloid fibers do not contribute to the detected oligomers. To detect an effect of the TasA:TapA ratio, we incubated 100 µM TasA (unlabeled) with 1, 2, 4 and finally 10 µM TapA (100:1, 50:1, 25:1 and 10:1, respectively) (Fig. 2*B*). The now overlapping monomer peaks decreased as the oligomer peaks increased in a TapA concentration-dependent manner. Interestingly, the maximum of the high molecular weight species shifts toward smaller S_20,w_ values with increasing TapA concentration (Fig. 2 *B*, *Right*, enlarged region) indicating the dominant occurrence of smaller oligomers. In summary, AUC shows that a fraction of TapA becomes part of the oligomers formed, and with higher TapA:TasA ratios on average shorter filament stretches occur.

The relevance of different TapA sections for promoting oligomer formation was investigated by using truncated proteins. Deleting the N terminus resulted in TapA_75-190_ that yielded only negligible amounts of oligomers in an AUC run with equimolar concentrations (Fig. 2*C*, turquoise curve; orange curve for comparison). By contrast, deletion of the C-terminal region 191 to 253 showed a very similar response (TapA_44-190_ in Fig. 2*D*, orange curve) to mature TapA (red curve in Fig. 2*B* and orange curve in Fig. 2*C*). Due to the large excess of TapA in our AUC experiments in comparison to in vivo concentrations [reported molar ratio of 100:1 for TasA to TapA (13)] and the fast depletion of monomeric TasA, we observed relatively “small” oligomers. In Fig. 2 *C* and *D*, the S_20,w_ values correspond to a distribution ranging from approximately dimers to octamers. Since a special role in TasA fibrillation was assigned to the N-terminal residues up to T57 of TapA by Earl et al. (15), we also investigated the effect of the adapted peptide C58SF61A-TapA_44-62_ on TasA. The mutation C58S was chosen to prevent the occurrence of disulfide linked dipeptides, and F61A to reduce hydrophobicity. A run with 250 µM of peptide (2.5 times molar excess over TasA) yielded a weak response (Fig. 2*D*, red curve), the effect being comparable to less than 4 µM mature TapA (Fig. 2*D*, green curve). This experiment corroborates the crucial importance of the TapA N terminus but also hints at a contribution of the upstream section 63 to 189 at least in vitro, with the subsequent C terminus being dispensable.

A complementary view of the oligomerization process is provided by detecting the disappearance of NMR signals originating from monomeric forms of TapA and TasA. Such signals may vanish due to unfolding or the formation of very large assemblies, which are not observable by solution NMR methods due to broadening of the resonance lines typical for large molecular entities. We followed the TapA-induced intensity change of TasA signals in the ^1^H chemical shift range between 0.5 and −0.4 ppm where folded proteins show characteristic, resolved signals (Fig. 2*E*, see arrows). Representative spectra of a 125 µM TasA/50 µM TapA solution recorded at different time points after mixing are shown in Fig. 2*E*, with the diagnostic TasA signal decreasing and the TapA signals remaining constant. Without TapA, the signals of TasA alone remain constant over days. A plot of the TasA signal decay at various TasA/TapA ratios, with monomeric TasA initially at 125 µM, is shown in Fig. 2*F*. Surprisingly, the intensity of the TapA signals remained constant, no decay or change of line shape was observed in all recorded series (see indicated TapA signal in Fig. 2*E*). At a TasA concentration of 250 µM the diagnostic TasA signal disappears within 10 h (Fig. 2*G* red curve) when 50 µM TapA is added (5:1). This higher sensitivity is exploited to investigate the effect of N-terminal deletions in TapA and TasA. Adding TapA without residues beyond E190 (Fig. 2*G*, orange curve) yields a wild-type-like response (red curve) at the same concentrations. However, deleting the C terminus of TasA (residues 240 to 261) slows the decay of the diagnostic TasA signal considerably (green curve), indicating a stabilizing effect. In agreement with the AUC experiments, the addition of 50 µM TapA_75-190_ to 250 µM TasA does not lead to a reduction of folded TasA (Fig. 2*G*, blue curve), supporting the notion that the N-terminal residues of TapA are critical for stimulating oligomerization.

An interesting picture emerges when looking at the AUC and solution NMR results together. At first sight, it seems surprising that according to AUC a fraction of TapA takes part in filament formation, but all of it remains as dynamic as a small protein since the line width of the TapA ^1^H-NMR signals do not broaden during these long experiments, as would be expected in the case of dimer and multimer formation. From this, we conclude that TapA cannot be localized within a filament, which is expected to be rigid, but must be attached in a flexible manner at one or both ends. Furthermore, the influence of the flexible C terminus of TasA (Fig. 2*G*, green curve) is intriguing, since it hints at an importance of this segment in the resulting filament. This and the fact that folded TasA monomers are converted to filaments raises the question as to the thermodynamic stability of the proteins involved.

### Monomer and Filament Stability.

To identify driving forces for the oligomerization process, we determined the overall stability of the monomeric proteins and of the oligomers formed via thermal shift assays (TSA) on selected TapA and TasA constructs, and on mixtures preincubated for 17 h at 20 °C. The melting of mature TapA is characterized by a biphasic profile with one peak at 50 °C and another at 64 °C (*SI Appendix*, Fig. S4*A*). Most stable is TapA_44-190_ with a *T*_m_ of 66 °C. In these cases, the TapA response is weak, whereas monomeric TasA shows a strong signal at a comparable concentration, and a melting point of approximately 43 °C (*SI Appendix*, Fig. S4*B*, blue curve). The solution of 100 µM TasA with 4 µM TapA (25:1, respectively) still predominantly contained monomeric TasA and TapA according to the AUC experiment (Fig. 2*B*), thus a melting point (43 °C) typical for monomeric TasA is observed (*SI Appendix*, Fig. S4*B*, orange curve). With 10 µM TapA, a shift to appr. 56 °C (*SI Appendix*, Fig. S4*B*, red curve) was detected. This increase hints at a higher stability of TasA oligomers (and filaments) than of the monomeric form. The stability of TapA-induced filaments of TasA_28-239_ is slightly lower, with a melting point of 52 °C (*SI Appendix*, Fig. S4*C*, orange curve). In summary, the process of TasA filament formation is thus driven by their higher stability, to which the C-terminal residues of TasA beyond K239 contribute significantly. The different melting points for monomeric TasA and the filaments may indicate structural rearrangements, whereas TapA has a much higher melting point than the filaments and is thus not expected to undergo large structural transitions involving its core domain.

### Structural Investigations of TasA Filaments.

**Fig. 3.**
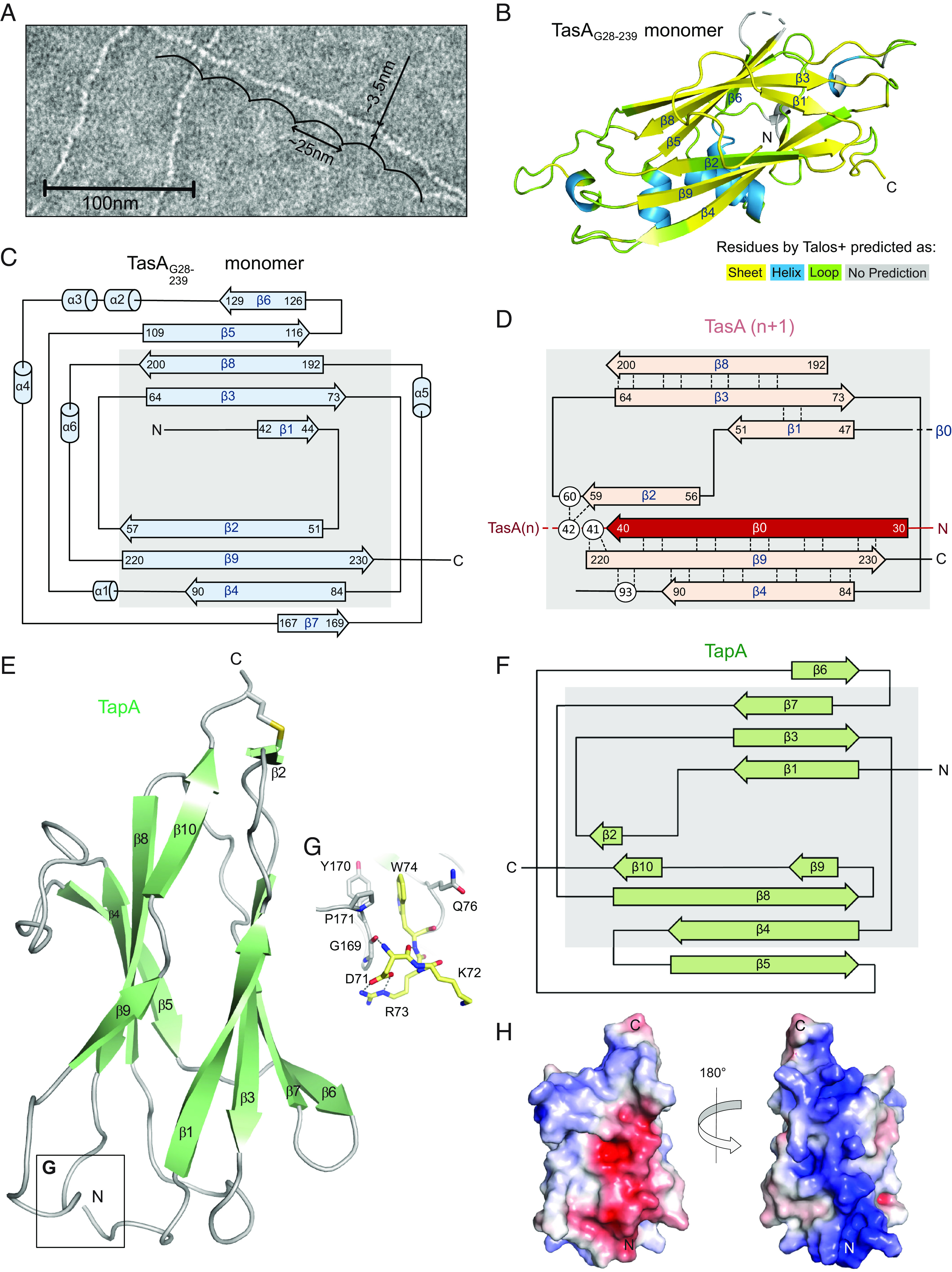
Comparison of the TasA monomeric and filament secondary structure. (*A*) EM negative stain of TasA filaments. (*B*) TALOS+ predictions of filament secondary structure plotted on the TasA_G28-239_ monomer X-ray structure (PDB 5OF1). (*C*) Secondary structure topology diagram of the TasA monomer structure shown in *B*. The gray box highlights the core region of TasA discussed in the text. (*D*) Hydrogen-bonding pattern identified by solid-state NMR on the basis of H^N^-H^N^ constraints within the TasA filament core region. (*E*) X-ray structure of TapA with disulfide bond (yellow) between C92 and C188. (*F*) Secondary structure topology plot of TapA_71-190_, revealing an Ig fold. (*G*) Detailed view of the region conferring stability to the N terminus of TapA_71-190_. The N-terminal residues _71_DGRW_74_ are shown in yellow and hydrogen bonds are indicated by dotted lines. (*H*) Electrostatic surface potential of TapA ranging from −5 kT/e (red) to +5 kT/e (blue) determined by the Pymol Plugin APBS Electrostatics tool. The left orientation is the same as in (*E*).

Further characterization of TapA-induced TasA oligomerization requires structural insight. TasA filaments were prepared by the two different procedures, either concentrating TasA at pH 7 over a membrane or induced by TapA, then harvested by ultracentrifugation. Identical morphologies were observed by negative stain EM, with a width of approximately 3.5 nM and a recurring oscillation of ~25 nM (Fig. 3*A*). A comparison of the ^1^H-^15^N correlation spectra of both types of filament samples shows almost perfect overlap (*SI Appendix*, Fig. S5*A*). Recognizable effects due to TapA are absent, in agreement with the solution NMR results. Backbone assignments (87% coverage, *SI Appendix*, Table S2) were obtained from standard triple resonance experiments (26, 27). The most important assignments are indicated in *SI Appendix*, Fig. S5*B*, and examples of sequential correlations between residues are given by means of strip plots in *SI Appendix*, Fig. S6. For sections with peak doubling, we included both chemical shift sets in *SI Appendix*, Table S2.

The assignment provides information on the secondary structure by interpreting chemical shifts. Backbone angles derived with TALOS+ [(28), a dihedral angle prediction program] indicate the overall conservation of the b-sandwich structure of monomeric TasA_G28-239_ (*SI Appendix*, Fig. S7 and Fig. 3*B*). In particular, helices are predicted at the positions where they occur in the X-ray structure, as are most of the β-strands (Fig. 3*B*, blue and yellow, respectively). However, the N-terminal region (residues A28-S41) which shows high B-factors in the crystal structure of the monomer (8) exhibits typical β-strand chemical shifts (*SI Appendix*, Table S2) in the filament. Furthermore, the chemical shifts of β2 are now typical for random coil arrangements (green in Fig. 3*B* and *SI Appendix*, Fig. S7).

Recording hNHH and hNhhNH experiments with through-space ^1^H-^1^H radio frequency-driven dipolar recoupling (RFDR) mixing (29) enabled us to observe cross peak patterns characteristic of hydrogen bonding in antiparallel β-sheets. Such pairs of hydrogen bonds lead to very strong cross peaks indicating interstrand H^N^-H^N^ contacts due to the short distance of 3.3 Å (*SI Appendix*, Fig. S8*A*), much stronger than those observed for parallel β-sheets (30). We detected such interstrand H^N^-H^N^-contacts involving the originally flexible, N-terminal segment D31 to S41 pairing with A230 to N220 (*SI Appendix*, Fig. S8*B*). These interactions are unequivocally assigned since several cross peaks in the 2D ^1^H-^15^N correlation are resolved with chemical shifts above 9 ppm (K35, A37, S41, I222, L224, A230, see *SI Appendix*, Fig. S5*B* where the hydrogen bonded residues are indicated in red, those in between in blue). Furthermore, the backbone signals of both strands are resolved in the 3D spectra used for resonance assignment (*SI Appendix*, Fig. S6), as are the correlations in the 3D hNHH spectrum (*SI Appendix*, Fig. S8*B*). The newly formed strand, termed β0, is now aligned with β9 instead of the segment N51-L57 that was interacting before as β2 (Fig. 3 *C* and *D*). Thus, β1 and β2 of the monomer structure undergo structural changes and the loop between them becomes a strand, now β1, that associates with β3, as indicated by β-sheet-typical cross peaks between the H^N^ of K49 and Q71, indicative of hydrogen bonding (Fig. 3*D*). Given the observed hydrogen-bonding pattern between the newly arranged β1 and β3 and the overall conservation of most of the TasA structure, the H^N^-H^N^-pattern involving G42, N59, and L60 (*SI Appendix*, Fig. S8*C*) must be intermolecular since G42 and N59/L60 are then situated at distant ends of the β-sandwich. Directly preceding G42 is β0 whose interactions with β9 must thus also be intermolecular. To detect this directly, we prepared a sample with two TasA preparations mixed (50/50), one being ^2^H,^15^N-labelled and ^1^H back-exchanged, the other ^13^C-labelled by supplying [2-^13^C]-glycerol to the growth medium. The deuteration of the first sample reduces the excitation of its naturally abundant ^13^C nuclei when cross polarization (CP) from protons is applied, and decreases relaxation of its ^1^H^N^. This approach reduces the detection of intramolecular H^N^-H^N^ contacts while intermolecular ones are promoted. We recorded 2D ^1^H-^15^N projections of a hchhNH experiment that first filters protons attached to ^13^C followed by a proton mixing period via RFDR prior to the ^15^N-^1^H correlation unit that selects protons attached to ^15^N. The resulting spectrum represents a selective subset of cross peaks indicative of intermolecular interactions (*SI Appendix*, Fig. S9*A*) that does not coincide with the largest cross peaks of the reference (*SI Appendix*, Figs. S5*B* and S9*B*). A superposition of both shows a large number of signals in the β-sheet region with ^1^H^N^ chemical shifts around 9 ppm (*SI Appendix*, Fig. S9*C*). A good coverage of the previously established intermolecular contacts is highlighted by the red labels in *SI Appendix*, Fig. S9*D*, except for G42 and L236, whereby the cross peaks associated with S41 and N220 are slightly shifted. Of course, more signals occur; however, it can safely be concluded that strand β0 inserts between strands β9 and β2. This yields the secondary structure diagram shown in Fig. 3*D*, where the interstrand hydrogen bonds detected by hNHH and hNhhNH experiments are indicated by dotted lines.

An additional structural rearrangement involves the C terminus. The signals associated with the amino acids beyond Q232 adopt considerably different chemical shifts in the filament than in the monomer (8) as evident from an extreme H^N^ chemical shift of 3.18 ppm observed for H256. A further stabilization of the C terminus through hydrogen bonds is indicated by H^N^-H^N^ cross peaks between S58 and V249 as well as between K65 and L236/I238 (*SI Appendix*, Fig. S8*C*) that are intermolecular since S58 and K65 are very distant to the C-terminal residues in the monomer. Indeed, except for L236 all signals of the C-terminal residues are involved in large intermolecular cross peaks in the mixed sample (*SI Appendix*, Fig. S9 *A* and *D* for assignments). This finding explains the higher stability of filaments formed by mature TasA in comparison to those formed by the shorter TasA_28-239_. A summary of detected H^N^-H^N^ contacts, including those observed involving signals of residues located in helices, are given in *SI Appendix*, Table S4.

Comparing the observed secondary structure and the style of strand insertion with examples in the literature, we found protomer connectivity and secondary structure in TasA filaments (Fig. 3*D*) to be similar to protein systems of the chaperone-usher pathway forming type 1 pili (31–34) on the surface of gram-negative bacteria. In fact, the unit defined by strands β0-3, β8, and β9 (Fig. 3*D*) resembles in its secondary structure pattern that of the respective Fim and Pap family proteins in pili, with identical donor strand (β0) insertion to complete a V-set Ig-like subunit [(35), Fig. 3 therein]. Still, other strand connections are different and reflect the original jellyroll fold of the TasA monomer structure.

### TapA structure.

The homology of protomer connectivity in TasA filaments with that in type 1 pili systems and our AUC results suggest a possible role of TapA as a chaperone-like facilitator of polymerization and raises interest in its structural properties, including both folded and unfolded areas. We therefore aimed to characterize all sections toward a comprehensive picture. The structure of TapA_44-190_ was first investigated by NMR, omitting the functionally silent C terminus predicted to be flexible. No long-range NOE pattern was observed involving residues 44 to 71, but a structure of the following Ig domain was obtained (PDB: 6QAY). To characterize the N terminus further, the variants TapA_54-190_ and TapA_60-190_ were investigated by 2D ^1^H-^15^N NMR spectroscopy and their spectra compared to the spectrum of TapA_44-190_ (*SI Appendix*, Fig. S10) in order to identify its signals. A number of strong cross peaks with random coil chemical shifts are observed in the latter spectrum that are not covered by signals of the shorter forms, corroborating the notion of a disordered N terminus.

Structures of initially TapA_75-190_ (1.28Å, PDB: 6HQC) and more recently TapA_71-190_ (1.07Å, PDB: 8AIF) were determined by X-ray crystallography (*SI Appendix*, Table S3). The longer construct was investigated because NMR showed long-range contacts between W74 and the core of the Ig domain. TapA_71-190_ shows a V-set Ig fold (InterPro code: IPR013106) composed of two antiparallel β-sheets (Fig. 3 *E* and *F*). D71 forms four hydrogen bonds involving residues G169, W74, and R73 (Fig. 3*G*). W74 and D71 together stabilize the loop between β-strands 8 and 9 (Fig. 3*G*). The additional hydrogen bonding suggests a higher stability of TapA_71-190_, which is supported by the TSA data (*T*_m_ = 60 °C for TapA_71-190_ and 46 °C for TapA_75-190_; see *SI Appendix*, Fig. S4*A*).

Structural integrity is supported by a disulfide bond linking C92 and C188. The section connecting the N terminus with the Ig domain contains two further cysteines (C58 and C68) that form an additional disulfide bond according to gel filtration data (*SI Appendix*, Fig. S11). Constructs with odd numbers of cysteines such as TapA_60-190_ and TapA_66-190_ showed a tendency to form dimers via disulfide bond formation with their free cysteine C68. Those dimers disappeared upon DTT treatment. TapA_44-253_, TapA_54-190_, and TapA_75-190_, on the other hand, appeared as monomers, since they contain even numbers of cysteines. However, as previously shown deletion of those cysteines has no functional effects on biofilm formation (14).

In summary, TapA consists of an Ig domain in its core (Fig. 3 *E* and *F*) and flexible N and C termini. However, the biphasic melting curve of mature TapA in contrast to the monophasic behavior of TapA_44-190_ observed in the TSA (*SI Appendix*, Fig. S4*A*) suggests an association of the negatively charged TapA C terminus (191 to 253) with the Ig domain, likely interacting with the prominent, positively charged TapA surface patch (Fig. 3*H*). Intriguingly, the secondary structure pattern of the Ig domain resembles in its core (gray background) that of the TasA filament structure (Fig. 3*D*) and the proteins of the chaperone-usher pathway (32). Fitting to this picture, the first 5 N-terminal residues of TapA,AFHDI, are nearly identical with the TasA N terminus (AFNDI). This gives TapA the potential to take part in the donor-strand mechanism toward the formation of the filaments.

## Discussion

Overall, the presented results suggest a mechanism of TapA-induced TasA filament formation in *B. subtilis* biofilms that resembles in its essence type 1 pili assembly in gram-negative bacteria, with TapA adopting a chaperone-like role reminiscent of the chaperone-usher pathway. AUC enabled us to demonstrate TapA to be necessary for filament formation but included in only small amounts (Fig. 2*A*). In a seemingly opposite way, solution NMR revealed fast, isotropic motions of the filament-attached TapA Ig domain (Fig. 2*E*) by the unchanged line shape of its narrow NMR signals after participating in oligomerization. Together with the observation of rigid, helical filaments by EM (Fig. 3*A*) this leads to the conclusion that mobile TapA can only be located at the beginning or end of a filament. Based on the near-complete (87%) assignment of the TasA filament backbone signals (*SI Appendix*, Figs. S5 and S6) obtained by solid-state NMR methods, the secondary structure of the filaments could be traced (*SI Appendix*, Fig. S7 and Fig. 3*D*), including the identification of intermolecular contacts (*SI Appendix*, Figs. S8 and S9). This revealed donor-strand insertion as the prime mechanism for oligomerization, with each TasA molecule donating its N terminus to become part of an Ig-similar core structure together with the second molecule (Fig. 3*D*). This process is driven by the higher stability of the filaments, as indicated by a melting point of 56 °C in comparison to 43 °C for monomeric TasA (*SI Appendix*, Fig. S4*B*). The X-ray structure of TapA in conjunction with NMR investigations revealed the presence of a complete Ig domain in the center of the TapA sequence and flexible N and C termini. The high melting point of TapA (64 °C) (*SI Appendix*, Fig. S4*A*), together with the fact that it already consists of a complete Ig fold, explains its inability to be located within a filament. Knowing the protein’s signal peptide cleavage site (Fig. 1) and the observed homology of N-terminal residues between TapA and TasA enables us to propose the mechanism of TapA-induced TasA filament growth, with TapA as an initiator (see below).

In detail, our AUC and NMR experiments demonstrate an acceleration of TasA filament formation by TapA in a concentration-dependent manner (Fig. 2). AUC experiments with similar concentrations of TapA (100 or 70 µM) and folded TasA (100 µM, Fig. 2 *C* and *D*) show multimers that range from approximately dimers up to octamers. At such high TapA concentrations, a high number of oligomers are initiated so that they remain small due to the limited TasA supply. TapA is incorporated into the oligomers generated, as demonstrated by the decrease of the TapA monomer peak in the AUC experiment shown in Fig. 2*A*, and the concentration-dependency of the oligomer distribution (Fig. 2*B*) at constant TasA concentration. However, in our time-dependent 1D NMR spectra of samples prepared with a TapA to TasA molar ratio of initially 1:5, TapA signals remain at the same intensity and do not show an increase of line width. Our AUC and NMR data also provide insight into the importance of the TasA C termini for filament formation and stability. The reduced NMR signal decay of TasA_28-239_ (*SI Appendix*, Fig. S5*A*) indicates slower oligomer formation and is paralleled by a decreased melting point of the resulting filaments (52 °C) in comparison to those resulting from wildtype protein (56 °C) (*SI Appendix*, Fig. S4 *B* and *C*). This supports that the stability of the filament affects the kinetics of oligomerization.

**Fig. 4.**
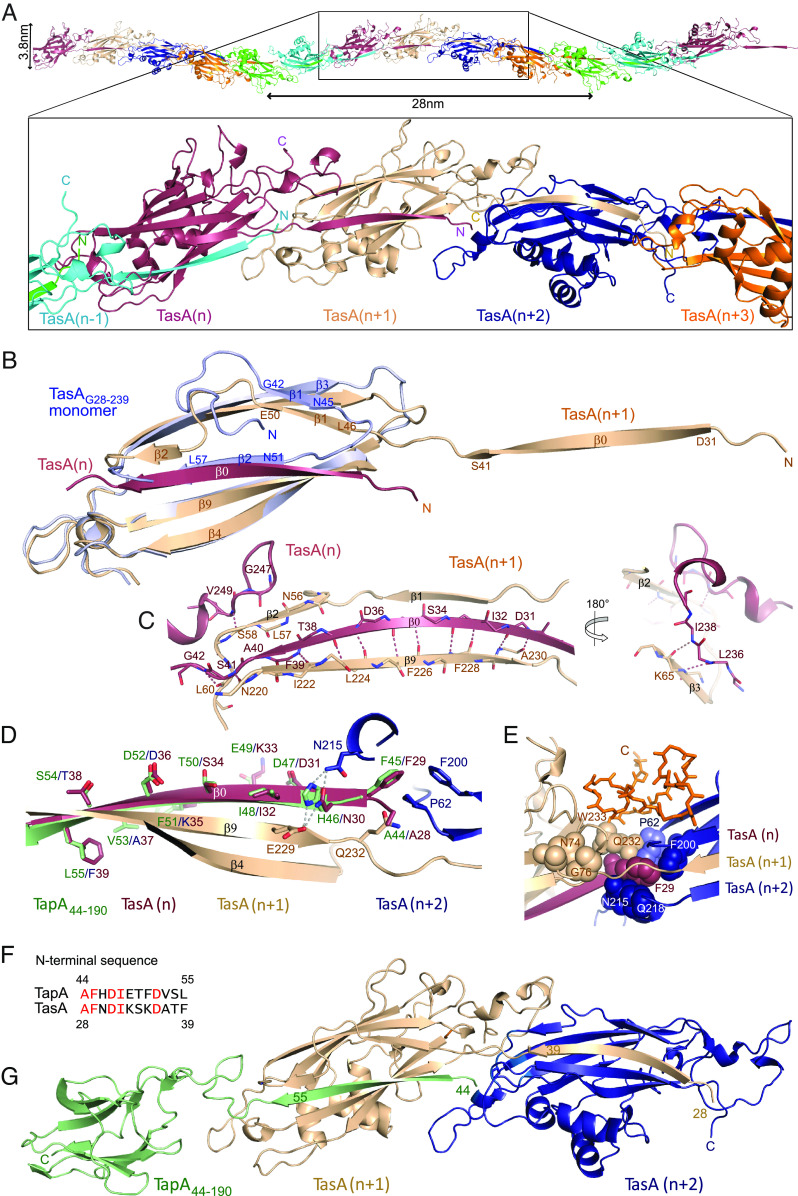
TapA–TasA interactions. (*A*) TasA filament AlphaFold model with close-up view onto five consecutive protomers. (*B*) Superimposition of the monomer X-ray structure (light blue) obtained by crystallization of TasA_G28-239_ with a protomer structure of the filament [beige (n+1)]. The N terminus of the preceding TasA molecule (n) is shown in red. (*C*) Hydrogen-bonding pattern identified by solid-state NMR confirming the β0-strand interaction. (*D*) TapA N terminus (green) superimposed onto the TasA β0 strand (red) of the TasA filament (*A*). Binding site residues F29, Q232, and F200 (TasA counting) form an ideal cavity for P62, all of them belonging to the most conserved residues. Side chain residues are presented as sticks and hydrogen bonds as gray dashed lines. (*E*) Close-up view on the TasA–TasA interaction site composed of the most conserved residues (shown as spheres), highlighting the binding pocket of TasA (n) F29 (red) surrounded by residues of TasA (n+1) and TasA (n+2) shown in beige and blue, respectively. (*F*) Sequence alignment of the N-terminal TapA and TasA residues. Identical residues are depicted in red. (*G*) AlphaFold model of TapA (green)–TasA (beige and blue) interaction.

Our solid-state NMR data obtained on filament samples allow for distinction of intra- and intermolecular interactions due to the conservation of most of the β-sandwich monomer structure and enable a comparison with an AlphaFold Multimer (24, 36) model of TasA filaments (Fig. 4*A*). The pitch and diameter as observed by EM (Fig. 3*A*) is reproduced by the AlphaFold model, which is in good agreement with the recently published cryo-EM structure (18). Our NMR restraints confirm these models (*SI Appendix*, Figs. S7 and S8), especially the detected hydrogen-bonding patterns (*SI Appendix*, Table S4) but also the chemical shift data that are in agreement with the secondary structure of the protomers (*SI Appendix*, Fig. S12). Surprisingly, nearly all of TasA in filaments is ordered and rigid according to the successful CP, whereas only 70+ residues contribute signals in CP spectra when fibrils are investigated (10). The Alphafold model and the EM structure diverge strongly in the region 117 to 126 for which we do not observe NMR signals, presumably due to high flexibility. Similarly, the spatially adjacent residues K176 and T177 discussed to be important for filament bundling (18) are likely flexible, however, G175 shows a ^1^H^N^ chemical of 9.6 ppm, which usually indicates an ordered structure. The signals of G175 are at the same position as in solution NMR spectra of the monomeric form (8), indicating a very similar chemical environment.

The difference between the protomer structure and our monomer X-ray structure of TasA_G28-239_ (Fig. 4*B*) is in β1 reversing its direction, and β2 now being shorter and parallel to β0, see also Fig. 3*D*. In accordance with the strong H^N^-H^N^ correlations shown in *SI Appendix*, Fig. S8, the N terminus of the preceding protomer n runs antiparallel to β9 of molecule n+1, β9^n+1^ (Fig. 4 *C* and *D*). The model reproduces the detected restraints involving the C-terminal residues well, see orange residues in Fig. 4*E*. It shows hydrogen-bonding interactions between residues 236 to 238 of protomer n and residues 64 to 66 of β3^n+1^ (Fig. 4 *C*, *Right* and *SI Appendix*, Fig. S8*C*) that manifest a short intermolecular parallel β-sheet, and a cation-π interaction between the W233 six-membered ring and H256 (37) that explains the extreme H^N^ chemical shift of the latter. Furthermore, the model shows a loop-like pattern for residues 250 to 252 and contacts between S58 of protomer n+1 and V249 of n (Fig. 4 *C*, *Left*). Altogether, these data indicate high reliability of the model concerning the TasA-TasA interface area. This is corroborated by the spectrum indicating intermolecular contacts in *SI Appendix*, Fig. S9. We have indicated example positions of 2D cross peaks expected from the evaluation of the AlphaFold model in *SI Appendix*, Fig. S9*A*, with good agreement. In its core, the interface is made up of residues from three protomers. β0^n^ spans two protomers, traversing molecule n+1 and touching n+2 via the aromatic ring of F29^n^ (Fig. 4 *D* and *E*). By binding β0^n^, a protomer n+1 is able to present an oligomerization-competent TasA-TasA interface to protomer n+2. In this context, F29^n^, Q232^n+1^ and F200^n+2^ form a perfect binding site for P62^n+2^, a “proline box” as known from the analysis of domains that bind proline-rich peptides (38) (Fig. 4 *D* and *E*). There, Q232 is involved in three hydrogen bonds with the P62 and F29 carbonyls, and F29 H^N^. Indeed, a ^13^C-^13^C correlation spectrum of a ^13^C,^15^N-labeled sample produced with [2-^13^C]-glycerol (39) shows a separated signal for the Cδ of P62 (*SI Appendix*, Fig. S13*A*), and cross peaks involving the Ca of A28 and F29 that indicate short distances (*SI Appendix*, Fig. S13*B*). Strikingly, this part of the protomer interface, the proline box, is the most conserved in TasA and camelysin alignments (see residues highlighted as most conserved in *SI Appendix*, Fig. S1), even though the identity of the compared sequences may be as low as 26%. The side chains of residues with highest conservation, together with a comparison of secondary structure, are shown as spheres in *SI Appendix*, Fig. S14, all appearing at the interface between protomers. As is common for β-sheet structures, strand β0 is not strictly conserved except for its beginning. The high conservation of the TasA-TasA contact area potentially enables the formation of joint filaments with TasA homologs from other species. This is in line with the observation that *B. cereus* is able to stimulate TasA production in *B. subtilis* (21).

With the data presented, a possible mechanism for *B. subtilis* biofilm formation emerges. According to earlier reports (2, 6, 13, 15) TapA is associated with the membrane. Analogous to the chaperoning of pili proteins (31–34), TasA molecules arrive unfolded through SecYEG and may start forming filaments with the help of the presented, homologous (Fig. 4*F*) TapA N terminus, leading to an initial heterodimer, then binding the next TasA molecule to become a trimer (Fig. 4*G*). The N-terminal residues of TapA (Fig. 4*F*) bind in a very similar way as TasA to protomer^n+1^, whereby H46 of TapA in position of N30 in TasA serves in the same manner to establish hydrogen bonds to the TasA residues E229^n+1^ and N215^n+2^ (Fig. 4*D*). Since folded, monomeric TasA supports biofilm formation when supplied to Δ*tasA^−^ B. subtilis* cultures (8), a scenario is required that explains the mechanism of its inclusion into the growing chain, driven by the higher thermodynamic stability of the filament (*SI Appendix*, Fig. S3*B*). Two pathways may be envisaged: unfolding of TasA or rearrangement of β1-2. The first option is supported by our observation that in filaments made from deuterated protein all ^2^H^N^ were always replaced by ^1^H^N^ after oligomerization, especially in the core of the protein (see assignment table in *SI Appendix*, Table S2). TapA thus functions as a chaperone in analogy to the chaperone-usher pathway since it already has a complete and especially stable (*SI Appendix*, Fig. S4*A*) V-set Ig fold and has the ability to complement TasA in the process of filament formation by donating its previously unstructured N-terminal strand. As a result, structurally similar TapA and TasA filament core units are observed (*SI Appendix*, Fig. S14 *B*–*D*). Owing to the stability of the TapA Ig domain, it is not able to open a cleft to receive a donor strand, and thus can only be located at the beginning of a filament, acting as filament inducer.

## Materials and Methods

### Constructs for Protein Expression.

Primers for cloning of different TapA constructs are summarized in *SI Appendix*, Table S5. Chromosomal DNA of *B. subtilis* 168 and primer pair 1 were used to amplify the gene for TapA_44-253_ (Uniprot-A0A6M4JJ23) by PCR. The PCR product was cut by XhoI and BsmI and cloned into pCA528 (40) (a modified pET24a vector with Kanamycin resistance) to generate pCA528_His_Sumo_TapA_44-253_. Further variants of TapA were generated by a modified QuikChange protocol for site-directed mutagenesis. For the C-terminally truncated version pCA528_His_Sumo_TapA_44-190_, a stop codon was introduced at position 191 with primer pair 2. Primer pairs 3 to 7 were used to generate additional N-terminally truncated constructs pCA528_His_Sumo_TapA_54; 60; 66; 71 and 75-190_, respectively. TasA constructs were prepared as previously described in ref. (8).

### Protein Expression and Purification.

Methods published previously for TasA (8) were applied to TapA.

### Prediction Methods.

Jpred4 was used to predict secondary structure (http://www.compbio.dundee.ac.uk/jpred4) (23). The probability for disulfide bridges was assessed by Disulfind (41). The ProteinDataBase (PDB, https://www.rcsb.org/) was screened for structures of proteins with similar amino acid sequence. Three-dimensional structure predictions based on the AlphaFold (24) algorithm were run via the publicly available ColabFold (https://github.com/sokrypton/ColabFold) (42) infrastructure through Google Colaboratory. For TasA oligomer structure predictions, the multiple sequence alignment was conducted with MMseqs2 (UniRef+Environmental) and model_type was set to AlphaFold2-multimer. Default settings were used for all remaining options.

### Strain Construction for Biofilm Experiments.

All primers and plasmids used are listed in the tables (*SI Appendix*, Tables S6 and S7). For the complementation of the P_tapA_-*tapA*∆13-234-*sipW*-*tasA*-kan in the *amyE* locus, the region was amplified by PCR from the genomic DNA of *u* 3610 comI^Q12L^ and cloned into the pBS1K plasmid by restriction digest with XbaI and SpeI, subsequent treatment with alkaline phosphatase and ligation. For the complementation of the different *tapA* constructs in trans in the *lacA* locus (plasmids pKXD2 and pKXD7), the full-length *tapA* as well as the truncated *tapA_1-190_* were amplified by PCR and cloned into pBS2E by restriction digest using XmaI and PaeI. The *tapA* constructs containing different deletions were constructed in a 2-step PCR and subsequently digested using XmaI and PaeI and cloned into pBS2E. The truncated TapA_1-57_ was constructed from pKXD7 using PCR amplification and KLD enzyme mix from NEB for circularization.

For construction of strain BKD30, the plasmid pKXD13 was transformed into BRK49. In short, the receptor strain was grown in competence medium (17.5 g/L K_2_HPO_4_, 7.5 g/L KH_2_PO_4_, 2.5 g/L (NH_4_)_2_SO_4_, 1.25 g/L tri-Sodium citrate x2 H_2_O, 0.5% glucose, 7 mM MgSO_4_, 0.1 mg/mL L-tryptophan, 0.02% casamino acids, 0.22 g/mL ammonium iron citrate) to OD_600_ 0.6 to 0.8, starvation medium (17.5 g/L K_2_HPO_4_, 7.5 g/L KH_2_PO_4_, 2.5 g/L (NH_4_)_2_SO_4_, 1.25 g/L tri-sodium citrate x2 H_2_O, 0.5% glucose, 7 mM MgSO_4_) was added for 1 h and then 1 mL competent culture was incubated with the DNA to be transformed for 2 h. Positive clones were selected on LB agar containing the antibiotics required and the integration verified by PCR and DNA sequencing. The different *tapA* constructs were subsequently transformed in BKD30 as described above.

### Biofilm Experiments.

Overnight cultures were prepared in LB-medium containing the required antibiotics (spectinomycin 150 µg/mL, kanamycin 10 µg/mL, erythromycin 1 µg/mL and lincomycin 25 µg/mL). The overnight cultures were diluted 1:100 in 10 mL LB-medium without antibiotics in 100 mL Erlenmeyer flask and grown at 37 °C, 180 rpm until OD_600_ reached 0.5. The cultures were diluted as described above in fresh LB-medium and grown again to OD_600_ 0.5. In a 24-well plate, 1.4 mL of medium optimal for lipopeptide production (MOLP) (30 g/L peptone, 20 g/L saccharose, 7 g/L yeast extract, 1.9 g/L KH_2_PO_4_, 0.001 mg/L CuSO_4_, 0.005 mg/L, FeCl_3_ × 6 H_2_O, 0.004 mg/L, Na_2_MoO_4_, 0.002 mg/L KI, 3.6 mg/L, MnSO_4_ × H2O, 0.45 g/L MgSO_4_, 0.14 mg/L, ZnSO_4_ × 7 H_2_O, 0.01 mg/L H_3_BO_3_, and 10 mg/L C_6_H_8_O_7_) (43) was inoculated with 14 µL culture and optionally 14 µL biofilm dye (final concentration 0.02 mg/mL Congo Red, 0.01 mg/mL Coomassie Brilliant Blue G250) (2) was added.

For biofilms on minimal salts glutamate glycerol (MSgg) agar plates, 10 µL culture were spotted on MSgg medium [5 mM potassium phosphate (pH 7), 100 mM Mops (pH 7), 2 mM MgCl_2_, 700 μM CaCl_2_, 50 μM MnCl_2_, 50 μM FeCl_3_, 1 μM ZnCl_2_, 2 μM thiamine, 0.5% glycerol, 0.5% glutamate, 50 μg/mL tryptophan, 50 μg/mL phenylalanine] (44) solidified with 1.5% agar.

The Biofilms shown were incubated at 30 °C 24 h for MOLP and 48 h for MSgg medium.

### TSA.

TSA was done in 50 µL setups in 96-well PCR plates (Biorad MLP9601) covered with a nonfluorescent sealing foil (Biorad INN120411) in a real-time PCR machine IQ5 (BioRAD) with a nonstandard filter combination (excitation at 438 nm, emission at 495 nm) to measure SYPRO Orange (5,000-fold stock from Invitrogen) fluorescence. A mixture of onefold SYPRO orange and protein in 20 mM phosphate buffer pH 7.0, 50 mM NaCl was heated 1 degree per minute between 25 and 90 °C. With unfolding the fluorescence increases and the negative first derivative shows the melting point as minimum (IQ5 software). The protein amount had to be optimized in advance; typically, the range from 5 to 50 µM was checked, depending on the size and structure of the protein. For comparable melting curves, 10 µM TasA_28-261_, but 50 µM TapA_44-251_ was needed.

### ThT Assay (ThT assay).

If not otherwise mentioned 100 µL 20 µM protein solution and 100 µL 40 µM ThT, both in 20 mM phosphate buffer pH 7.0, 50 mM NaCl, were mixed in a black 96-well plate with clear bottom (Corning 3615), and fluorescence was monitored using the bottom mode (excitation at 438 nm emission at 495 nm; 5 nm band width, gain 80) in a Safire microplate reader (Tecan).

### Solution NMR.

All NMR spectra were recorded on Bruker AV-III spectrometer operating at 600 and 750 MHz (^1^H frequency) at a temperature of 27 °C. In both spectrometers a “TCI”-type cryoprobe equipped with one-axis, shielded gradients was used. The spectrometers were operated using TopSpin 3.5. For the determination of the structure of TapA_44-190_ a sample labeled with ^13^C and ^15^N was used, the concentration was 540 µM in 20 mM phosphate buffer pH 7.0, 50 mM NaCl. An extensive set of experiments was recorded [^15^N-HSQC, ^13^C-HSQC, HNCA, HNCACB, HN(CO)CACB, HNCO, HN(CA)CO, HBHA(CO)NH, H(CCO)NH, CC(CO)NH, HCCH-COSY, HCCH-TOCSY] resulting in the assignment of almost all resonances of aa 75 to 190. Distance information was extracted for that region from 3D ^15^N- and ^13^C-NOESY spectra. In all spectra, water-suppression was accomplished using a Watergate scheme. Data were processed using topspin and analyzed using the program from the Collaborative Computing Project for the NMR community (CCPN), CcpNmr Analysis 2.4.2 (45). The structures were calculated using ARIA (46). For the analysis of the dynamics of the protein ^15^N-T1, T2, and hetNOE spectra were recorded using a ^15^N-labeled sample of a concentration of 1 mM, again using Watergate for water suppression. The assignments are deposited in the Biological Magnetic Resonance Data Bank (BMRB) (entry 34341), the structure can be found in the PDB (code 6QAY). To check samples and to compare protein constructs of different length, ^15^N-HSQC spectra were recorded using Watergate water-suppression. Experiments to follow the effect of TapA on TasA were recorded as one-dimensional spectra using Watergate with excitation sculpting. To follow the development over a longer time period, pseudo two-dimensional spectra were recorded. The data were processed using topspin 3.5, and decay curves were extracted using in-house scripts.

### Filament Formation.

Monomeric TasA separated by gel filtration in 20 mM phosphate buffer pH 7.0 with 150 mM NaCl was slowly concentrated over 2 to 3 d in an Amicon stirring device equipped with a 10 kDa regenerated cellulose membrane at 10 °C. As a result, a turbid solution was obtained. Prior to sample transfer into the NMR rotor, the filaments were sedimented by ultracentrifugation for 1 h at 8 °C and 130,000 g, using a TLA110 fixed angle rotor.

Alternatively, growth of TasA filaments (e.g., 250 µM) was achieved in the presence of TapA (e.g., 50 µM) overnight at 20 °C in 20 mM phosphate buffer, pH 7.0 with 150 mM NaCl. The deuterated sample used for establishing resonance assignments was prepared as described below under “Analytical and Preparative Ultracentrifugation”, last paragraph.

### Solid-State NMR.

Assignment spectra were recorded on a Bruker Avance III spectrometer operating at 600 MHz ^1^H Larmor frequency. For studies on TapA-induced filamentous TasA, fully ^2^H, ^13^C, ^15^N labeled and 100% ^1^H back-exchanged protein in 20 mM phosphate buffer, pH 7.0, and 150 mM NaCl, washed with the same buffer but 50 mM NaCl, was spun at a 60 kHz magic angle spinning (MAS) rate using a 1.3 mm rotor. Temperature control was achieved with a BCU II, a gas flow of 1,200 L/h, and a cooling gas temperature of 252 K. For the assignment spectra, manufacturer provided pulse programs hNH2D.dcp, hCaNH3D.tcp, hCONH3D tcp, hcaCbcaNH3D.tcp, hCOcaNH3D.tcp, hcaCbcacoNH3D.tcp, and hcoCacoNH3D.tcp with CP (47) for heteronuclear magnetization transfer and a J-coupling based scheme for homonuclear (^13^C-^13^C) transfer (48) were employed (26). Parameter recommendations provided in the pulse programs were closely followed and subsequently optimized for existing conditions. Spatial information was extracted from in-house programmed hNHH and hNhhNH pulse sequences. The hNHH consists of an hNH CP step yielding the two indirect dimensions followed by a 1.5 ms ^1^H-^1^H RFDR step (29) prior to acquisition. The hNhhNH pulse includes an additional hNH CP step.

For the sample with mixed labeling pattern, we prepared TasA monomer solutions that were either ^2^H, ^15^N labeled or ^13^C labeled by supplying solely [2-^13^C]-glycerol as carbon source during expression. These solutions were mixed 50/50 and filaments created by concentrating the sample in aqueous buffer with 20 mM phosphate buffer, pH 7.0, and 150 mM NaCl. Filaments were washed with the same buffer, however, containing only 50 mM NaCl, and transferred into a 1.3 mm rotor. For the measurements, NMR spectrometer and settings were as above. A ^1^H-^15^N spectrum (hNH2D.dcp) for comparison was acquired optimizing the previously determined parameters. A hchhNH pulse sequence was used to monitor intermolecular contacts. It consisted of two CP blocks (hch and hNH, respectively) in between which a 3 ms RFDR mixing period was introduced (180 rotor cycles at 60 kHz MAS, depiction in *SI Appendix*, Fig. S9*E*).

For ^13^C-^13^C correlation measurements by dipolar-assisted rotational resonance (DARR) either uniformly ^13^C,^15^N- or sparsely [2-^13^C]-glycerol, ^15^N-labeled TasA was purified and subjected to filament formation through concentration as mentioned previously. TasA filaments were filled into a 3.2 mm rotor and measured at a Bruker Avance III spectrometer operating at 800 MHz ^1^H Larmor frequency. For uniformly labeled TasA, a short mixing time (20 ms) was used and for the sparsely labeled protein a long one (400 ms).

Data acquisition and processing was conducted using TopSpin 3.5pl7 (Bruker). Peak picking and protein assignment was conducted with CcpNmr Analysis 2.4.2 (45).

### Crystallization and Structure Determination.

TapA_75-190_ and TapA_71-190_ in 20 mM Tris buffer pH 7.0 50 mM NaCl were crystallized using the sitting-drop vapor-diffusion method. Crystallization setups were performed by using a Gryphon pipetting robot (Matrix Technologies Co.) for pipetting 200 nL protein with a concentration of 23 to 26 mg/mL to an equal volume of precipitant solution. The Rock Imager 1000 storage system (Formulatrix) was used for storing and imaging of the experiments. Crystals appeared within 1 to 3 d for both TapA variants and were flash-frozen in liquid nitrogen in the presence of 20% ethylene glycol. For crystallographic phase determination, SeMet-incorporated TapA_75-190_ was crystallized in a crystallization condition containing 20% MPD, 0.1 M Bicine pH 9.0, whereas native TapA_75-190_ was crystallized in a 20% PEGMME 550, 0.1 M NaCl, 0.1 M Bicine pH 9.5 condition. The N-terminally elongated TapA_71-190_ yielded crystals in 24% PEG3350, 0.35 M LiNO_3._ All diffraction data were recorded on BL14.1 at BESSY II (Helmholtz-Zentrum Berlin, HZB), processed and scaled using XDSapp (49). The crystallographic phase problem for the SeMet derivative of TapA_75-190_ (1.3 Å data, anomalous signal up to 1.6 Å) was solved by using the automatic crystal structure determination platform Auto-Rickshaw (50). The structure of native TapA_75-190_ (1.28 Å) was solved by molecular replacement with Phaser (51) using the SeMet-TapA_75-190_ Auto-Rickshaw structure as the search model. The TapA_71-190_ structure was also solved by molecular replacement with Phaser, but here the TapA_75-190_ structure was used as the search model. Finally, both protein models were manually built using COOT (52) and iteratively refined using Refmac (53).

All residues of the TapA_75-190_ structure are nicely explained in the electron density. Crystals of TapA_71-190_ contain 3 molecules (chains A-C) per asymmetric unit. Chain A covers residues 72 to 189, chain C residues 71 to 188, and chain B shows the complete molecule. The missing residues in chain A and C were disordered and therefore not visible in the electron density.

All structure statistics is given in *SI Appendix*, Table S3. 95.6% of the residues in the TapA_75-190_ structure were in the allowed regions of the Ramachandran map and 96.6% for the TapA_71-190_ structure. The Ramachandran statistics of both structures were analyzed using Molprobity (54). Figures and structure superimpositions were generated with PyMol (http://www.pymol.org). The atomic coordinates of TapA_75-190_ and TapA_71-190_ have been deposited in the Protein Data Bank (PDB ID codes 6HQC and 8AIF, respectively).

### Analytical and Preparative Ultracentrifugation.

Sedimentation velocity experiments were performed with a Beckman Optima XL-I analytical ultracentrifuge at 20 °C and a rotor speed of 40,000 rpm (129,000 g at cell bottom). The samples indicated in *SI Appendix*, Table S1 were measured in dialysis buffer (20 mM Na phosphate pH 7.0, 150 mM NaCl) separately or in combination at a final concentration of 100 µM unless otherwise stated. In titration experiments, the TapA concentration was reduced to 10, 4, 2 and 1 µM. Interference data with a total of 200 scans were recorded every 5 min. Sedimentation coefficient distributions c(s) were analyzed with the program Sedfit (55). The protein partial-specific volume and the buffer physical constants were calculated from amino acid and buffer composition, respectively, using SEDNTERP (56). Figures were created with GUSSI (57). After the run the samples have been recovered, mixed, and applied to ThT assay and TSA as described there.

To obtain samples for NMR and ThT assays (Fig. 2*A*), the mixtures of 1.5 mL of 100 µM ^2^H,^13^C,^15^N-TasA, and 1.5 mL of 100 µM unlabeled TapA were ultracentrifuged (Beckman Optima Max) under the same g-force as mentioned above overnight. The resulting pellet was washed once with 20 mM Na phosphate pH 7.0, 50 mM NaCl and filled into a 1.3 mm MAS rotor for NMR. A small amount of sample was analyzed by negative stain EM.

### ITC.

ITC experiments were performed using a PEAQ-ITC microcalorimeter (Malvern). All titrations were performed at 18 °C with 200 µM TapA variants (500 µM for TapA peptides) in the syringe and 20 µM TasA in the reaction chamber. The protein as well as the titration components were dissolved in buffer containing 20 mM Tris/HCl and 50 mM or 150 mM NaCl, pH 7.0. Malvern software was used to visualize the binding process.

### Sequence Alignment.

Additional protein sequences homologous to TasA were located by supplying diverse sequences annotated as camelysines to the program blast; evolutionary relevant sequences were chosen from the result. Alignment was then performed with MEGA11 (58) and the ClustalW algorithm (59) with default settings. The resulting alignment was then exported to .fasta format and visualized using the strap (60) web application (https://www.bioinformatics.org/strap/aa/).

## Supplementary Material

Appendix 01 (PDF)

## Data Availability

TapA structures are available in the PDB under accession codes 6QAY (61) for NMR, and 6HQC (62) (TapA_75-190_) and 8AIF (TapA_71-190_) for the X-ray structures. Solution NMR chemical shifts of TapA are deposited in the BMRB under entry 34341 (63). Solid-state NMR raw data, chemical shift and peak lists of TasA filaments are deposited in the BMRB as entry 51785 (64). Processed TasA filament spectra and the CcpNmr Analysis 2.4.2 (45) assignment project are uploaded to zenodo (10.5281/zenodo.7534572) (65). All study data are included in the article and/or *SI Appendix*.
