# Supplementary material for: TapA acts as specific chaperone in TasA filament formation by strand complementation
Source: Proc Natl Acad Sci U S A. 2023 Apr 17;120(17):e2217070120. doi: 10.1073/pnas.2217070120 (PMC10151520; doi:10.1073/pnas.2217070120)
Supplement: Supplementary file 1 — Appendix 01 (PDF) [file pnas.2217070120.sapp.pdf]

PNAS

Supporting Information for

TapA acts as specific chaperone in TasA filament formation by strand complementation

Yvette Roske, Florian Lindemann, Anne Diehl, Nils Cremer, Victoria A. Higman, Brigitte Schlegel, Martina Leidert, Kristina Driller, Kürşad Turgay, Peter Schmieder, Udo Heinemann, Hartmut Oschkinat*

*Corresponding Author: Hartmut Oschkinat
Email: oschkinat@fmp-berlin.de

This PDF file includes:

Figures S1 to S14
Tables S1 to S8
SI References

Supplementary Figures

```
                                                         29            42                           62
1  TasA  B. subtilis (strain 168)|P54507          MGMKKKLSLG.VASAALGLALVGGGTWAAFNDIKS.KDATFASGTLDLS.......AKENSASVN..LSNLKPGD........
2  CalY1 B. cereus (clone B905)|A0A0A3VYJ9        MSLKKKLGMG.VASAALGLALIGGGTFAFFSDKEV.SNNTFAAGTLDLTLN.........PKTLVDIKDLKPGD........
3  CalY2 B. cereus (clone B905)|A0A0E3SV09        MTLKKKLGMG.ITSAVLGAALVGGGTFAFFSDKEV.SNNTFAAGTLDLALN.........PSTVVNVSNLKPGD........
4  Thermococcus gorgonarius|A0A2Z2M7U5              MKSIAVA.LLLIGLVFAGAGYGTWAIYQDTETSEGNSIELGTLDLVLTDG.....VPVANGQWFIPNAVPGS........
5  Bacillus mycoides|C2Q5K3                 MIWEEQKMSLKKKLGVG.VVSAALGLSLIGGGTYAYFSDQVV.TNNSFAAGTLDLAMQ.........PTTSLNLENLKPGD........
6  Bacillus thuringiensis|K0G0I4                  MSLKQKVGMG.VVTATLGLSLTCGGVFAYFSDSET.SSNSFQAGTLDLSIN.........PSVIVDVKDLKPGD........
7  Haloferax gibbonsii|A0A871BKP0             MPEKVFKLTRRRALGGLLTIGAGSAAAGVGTFALFNDTESSTENTLQAGTLSLADG.........ASSASFSATGLAPGDE.......
8  Paenibacillus graminis|A0A089M0B1              MGIKKTLGFG.IASAALGLSLIGGGTFAYFSDTAT.STATFAAGTLDLNSD.........PSVIVDIQNLKPGD........
9  Aquisalibacillus elongatus|A0A3N5B9I6          MDIKKKLGIG.VLTGALGLSLVGGGTWAAFNDVEE.TTNVFAAGVLDLE........IGEESTMDFSLDNLKPGD........
10 Bacillus oleivorans|A0A285CS81                 MSLKKKMSMG.ILSGALGVSLIGGGTWAAFNDVEV.VNNTLAAGTLDLV........VGENTTMNFTLSNLKPGD........
11 Aureibacillus halotolerans|A0A4R6UFZ6          MGLKRKLATS.VMSASLGLSLVGGGTWAAFNDVEN.IENTFAAGTLDLAVI.....TKDKSNSVFK.LLNMKPGD........
12 Bacillus sp. 1NLA3E|N0ATI4                     MSIKRKLGLG.AASAALGLSLIGGGTFAYFSDAAT.LHNGFAAGTLNVELQK......AWDFPLNFDLSNIQPGD........
13 Blautia luti|A0A564VQ52                      MMKNRKLVKGI.ALAAVIT.ALAAGGTAAYLTDFET.ATNSFTVGKVDIDLD........EPNWKPEDNTDLVPTQ........
14 Bacillus oleivorans|A0A285CTH3                 MSLKKKLTMG.ALSATLGMSLVGAGTWAAFNDIEE.VNASLAAGSLDLV.......VDEYNGPVNFNISNLKPGD........
15 Paenibacillus taihuensis|A0A3D9SB88            MSIKAKLGMG.IITASLGLSLIGGGTYAYFNDAGSSIQNSFASGTINLNATK......AWDFPANFDLVNMKPGD........
16 Neobacillus mesonae|A0A3T0I131                 MGIKKKLGLG.MASAALGLSLIGGGTFAYFSDEAT.IHNGFAAGTLNLEVGK....YPGTQWPVNFDLSNLRPGD........
17 Methanosarcina barkeri|A0A0G3CB53               MANKRVLVS.ILALGIAVTLAGAGTWAAFQDTETSTGNTFSSGTLDMKLSNDGSTYTDGVTATWTSPANFKPGD........
18 Lachnospiraceae bacterium|A0A7G9GBU6           MKRG..TKTI.MLAAALAGVLIVGGVSAYFTDADT.ATNTFTVGKISLDLQ........EPSWNPDQVKDLTPNQ........
19 Virgibacillus salinus|A0A1H1GBR8               MNIKKKMGTV.ILAGALGLSLVGSGTFAAFNDVEK.IDNTFTAGTLDLV........LTGQEKTSFEFSNLEPGE........
20 Sporosarcina sp. resist|A0A7G8X4J2             MGIKQKLAMG.IATGALAVSMIGGGTYAYFNDVET.STNTFAAGTLDLSLK.........PETIIDVKNIKPGD........
21 Neobac. massiliamazoniensis|A0A0U1NYG4         MKIKTKLGMV.AASGALGLALISGGTYAFFNSKVE.ATSTFEMGTLDLSVN.........PSTIVSVKNLLPGD........
22 Piscibacillus halophilus|A0A1H9E238            MSIKKKLGMG.VLSAALGISLIGGGTFAYFSDTEE.TNNTFAAGTLDLSVD.........PTTVIDVDNIKPGD........
23 Hungatella hathewayi|A0A174ECC6                MRRNYRKASQIVSLGALAIVLIIGGTLAYFNQDLS.ASNVFLTGKYDTDLH..........EEFKPPGDWQPGV........
24 Alteribac. persepolensis|A0A1G8JRA6            MKKTKKALIATTLAGAVAVGAG.FGTFSWFTDSQS.TDGEITAGTLTLNDLS.........EYTVFSEGNMQPIDPEITPDLI
25 Bacillus velezensis|A0A8F9SKQ0                 MGMKKKLSLG.VASAALGLALVGGGTWAAFNDVKS.TDATFASGTLDLS.......AKEQSANVN..LSNLKPGD........
26 Psychrobacillus sp. OK028|A0A1G9SR36           MSIKQKLAMG.ILTGGLAISMIGGGTYAYFNDVEV.NNSSFAAGTLDINVAG......NDADNAIINVTNIKPGD........
27 Bacillus pumilus|A0A8G1J983                    MAKKRSIRLG.VLSGALGLALIGGGTWAAFNDVEK.ANAVYSTGELDLS.......AKENSGAIN..LANLKPGD........
28 Haloechinothrix alba|A0A238Y1I1                MRIEKKTAVG.VAAAAAAALAITGGTFATFSDTEEAPNPSITAGTLDLTTG.......GEEQSESFAIEDVAPGYSSEE...A
29 Clostridium cf. saccharolyticum|D6DLJ6           MKYYKQLG.IILAGGVLAVGISATWAYFSDRND.IANPFHTSNSAIDMI...........ENYNPAETVLPGE........
30 Salimicrobium halophilum|A0A1G8S890            MSVKKKLAVG.VMTGALGLSLVGGGTLAAYNDVAK.VNNHFASGELNLEV......GDSHNKAISFDLTNMKPGD........
31 Bacterium BMS3Abin01|A0A2H6K5U5                  MNKRILIS.VLAVSLLAMLAGAGTYAYFSDSETSSGNSFAAGTLDLKVADNDE.GYGDGVSQTWVIGNMVPGVS.......

            74
1  KLTKDFQFENNGSLA.IKEVLMALNYGD..FKA...........NGGSNTSPEDFLSQFEVTLLTVGKEGGNGYPKNIILDDANLKDLYLMSAKN..DAAAAEKIKKQIDPKFLNASGKVNVATIDGKTAP
2  SVKKEFLLQNSGSLT.IKDVKLATK.YTVKDAKGD.............NAGEDFGKHVKVKFLWNWDKQSE......................PVYETTLADLQKVDPDLLAK.DIFAPEWG.......
3  TIEKEFKLENKGSLD.IKKVLLKTD.YNVEDVKKD.............NK.DDFGKHIKVTFLKNVDKHET......................IVKQTTLDKLKGDTLTAVDN.DLSAWFWD.......
4  LYNGDIKIINNGTVD.ADHVEIAIELVCWEDDDGNIDNGRAWGPEPDEVNGVGDLPQEIKVNMQYREEWIG...........................PSATLQLENGAALGDVVYNVYNLPAPPRVNG
5  KILKKFNLTNSGTLD.IKDIMMKID.YTVNDLKQN.............NTTEDFGKHIKVQFLLDWDPAKSP......................VYETTLAELKSQSPEIASKKVFYSKWTET......
6  FIEKNFTLENKGSLD.IAKVALETS.YEVTDAKLN.............NDGEDLGDHIIVKFLVNDGKPSNPNDDHE.................ILWETKLSKLKTMKPEDVSTSLERHNLID.......
7  TDTFEVDLENDGTLDGSLDIDLALTENDSDATAGDD.........GDADASVADLAGVLDVTTFEVG................................SLDVLADRFGGSATLADVVANNAGED..DDS
8  TVTKTFKLKNDGTLD.IGSILLKTT.TEVTDTLGD.............NNGADLSKFIKVKFLKNNDKGTIISP.................EVVVYETTLDALKGQTPDLVNN.TGWDQILT.......
9  HWTETLVLRNNGTLD.INQILT.SIEVTG.WEDVDALDLNSKVYDGAGNNKLSDFLKQFEVTVT....................................DGDGNV.FFDGTLNQLHGQSNID.ITGT
10 YFNKSLVLTNGGSLD.INQILVHANKLAG.WTDKDVLDLNTEIGANSGNNSEDDFLSQFKVTITKL.................................AVDPANNVDVFDGTLADLVAGISVDELTDT
11 SKTAEFELKNDGSLA.IKEVLLDITVPEDGFKNGEN...EYVNEHGGEENEAIDFLEQFQVEILRIDEETG..............YDFTLIEEKD..GVTLADFAKKDLPAEIETVDGAINLAPIH.TERP
12 SFERQFVLKNSGSLA.IEDVFMGVT.ANVTNPLGTG............GTSDQFLDALNVKYFVDATPPYIDPDAPDA.............GYLLINSQDISLREAIAGDFAG.KIKADYIKNG.KLNL
13 VIRKDPYVANKGVNE.AFVYLEVSVPVRNVITAAK............DGTRNALAKTELFSFTKNKDWTQL.........................ERTEVGQNMVYTYAYN.....HILKPGTKTTT
14 TMTRYIKLDNAGTLA.IKDVLLSIDSVN..FTDYLP...VGADSDIYGANTADEYLSQFKVTLLKTGIEGADEEIISS.ADNITLADIYLATNPDQLDPVAIAKLSAAIG.NGHWVDGHVNLASAA...QN
15 VTERQFVLQNIGSLD.IANTLMSFT........SDA............GSDAGLLNTLTISYFVSVGNN........N..............EYLLMNSQDITLSDAIAGNYAG.KIKAEYLTADGKLNL
16 TVERTFDLKNVGSLA.IEDTYLDFSKVSVENPLNTG............ATDDDFLSALSVTYFYETVVN..GQYVPES...............MLVNSQKISLKDAIAGNYVG.KIQPRFLKGT.KLNL
17 TFGNELRFTNVGSVN.INHLYMYPEDLTNSGGTGG...........IKLSDKIIITKVTGHITRDGSNLVWG..................NRADFIGSVVGNRDGTLTLTEFCSAKFVAYCLSPIIFG
18 VVQKDPQIKNDGIND.EFVFLEVTVPYKNVVTVND............AGVKQTAADTELVKYTVNAGWTELG.......................SGEKDTVAETVLHRYVVYGTDQACTALAKDQVTPA
19 SFTKTITLKNNGSLA.IKEILMSSMAIEGSWIDKT....NQKLPDN.GMNTVDDFLEQFNVIVN........................................QGETEVFNGTLADLNSG...KVIMGS
20 WMNRDFTLKNDGTLD.ISKVLLKTE.YTVTDKNGD..............NVENFGSHIRVNFMWNHDKSITGPWTGL..............DNVVYQTTLAELQGMTPDAVAN.KVFVPYLE.......
21 YMTRSFNLKNTGTVD.IAKVLLKTE.YTVTNKDGAP.............ANTDDLGKHIRVNFLFNQDKAALGTWLPE..............DQVVFQTTLYDLQNMTPDAVEN.RIFHDHLE.......
22 VMVREFELINGGTLD.ISSIDLSTA.YEVIDAEGN..............NTDDFGKHIKVLFLENADKTGDGWIIGDY..............NDVIAETTLYDLQNQTPDAVENVQGFFTHLLGL...NG
23 EIPKKVDIKNKGNVDVVAVARMTESCVRKEDVFITTYETVD.GRKTERQEKVASKGDVLPLQFEDSDGTQQEFALKNFG......SDVVPYAEAKSPEEYRNKWVYTYDENSKAYYFIYMGLIEGGNTSPG
24 TEGENITVENTGSLEMVPRLQLDVEIKDDKGNDAS................ETLSDEYMIKMTFDR..................................NDMNWSEEISADKVIDNRGFVDPDWFPSD
25 KLTKDFEFRNNGSLA.IKEVLMALNFTD..FKG...........AKKGNESAEDFLNQFEITVLTVGKEGGNGYPKNIILKAASLKDLYLMSTKQ..DKAAAEAISKHIDPKFLSESGKVNVATINGKTAP
26 TMLRTFKLNNTGSLD.VSKVLLTSK.YTVVDAKGSD............NGGADFGEHIKVKFLINKDKRTE......................VVHETTLKDLS..KTDVTDR.DILGWILDG......
27 RIKKEFHFENKGSLA.INQVLMSLDYSQ..FQDG.......ASAKNGGKNTAEEFLSQFQVSVLTVGAEGGNGYPKNIILDHANLLDLHQLTSKQ..DQTAFEKLRHAVDEKFLHESGKINVATVDGTVAP
28 GEGKTIQYENEGSIDGDLTLTVDYSTDEVGCNDPE.............IDAEKAFYDDESIEDCGN...............................DTELDEQLYAEIIGPDGTVAGEDLVGHLKG
29 TVEKEPYFVNQGSTDLVLRLKMDTYWIDQNGNRKD.................DLGGAYIFGGLQPEEDD..............................YVTLYLPDGWDDDWVQIEDYYYYKHILPG
30 NVQRIFTLNNIGSLA.IKDVLMEGSAGAF.............SDLDGDSNKNGFLKQFEIDFMQVDGESNQWEP..........REDVVMSGKT...LTLHDVVHGNTTSKVNSSHLVGNKINLASVAAG
31 SVTNSMSLQNAGSVN.ADHIELGFSYSIDETSNPVES......DTNPASAPGDMARMIEITAMTYDGVN.........................FATSFVDANGNGWFDLEDLSLPPYSSSGG.YLDN

                                          215              232
1  EYDGVPKTPT.DFDQVQMEIQFKDDKTKDE...KGLMVQNKYQGNSIKLQFSFEATQWNGLTIK.KDHTDKDGYVKENEKAHSEDKN
2  EKGGLAAGT...EDYLWVQFEFVDD..........GKDQNIFQGDTLNLEWTFNANQEAGEEK
3  EKG.ISAGK...SDKFKVKFEFVDN..........GKDQNQFQGDKLQLNWTFDAQQTAGEER
4  DVNDALWNKK..YGQFDMYLWIEDT..........GQPQNYWQGDVCEIIVHVALAQDSSQQILSPYGFTEYPVPVLPE
5  ..GGLKPGK...MDWFWIKFVFEDN..........GTDQNVFQGDSIALKMEFQANQTEGQER
6  ...GITSGE...TDYLHVLFSFEDN..........DQDQNKFQGDSLQLNWTFHAEQTKGVEK
7  NVRDIALASG.ETKTFSFALEMQES............AGNEFQADGLDIQFDFELNQRDSQ
8  EADGIKAQS...SDSFTVKFEFVDT..........GEAQNYFQKDKLKLTWNFEAKQGPKKDY
9  DTNTVGLSVN.DNMTYQVDFKFKNVDETFDG..SRFQVQNKYQDEQAEINVKFEATQMPGEEQS.......NGWAN
10 DATTVGLASG.AQQIYNVYVEFVEDSTTFPG..SRLHVQNKYQDEMSNLEFVFEATQMPGEDRS.......ND
11 EYTGLPVNPL.DDEVVKIKIVFVEDDTIDDS.VPGEYLQNRYQGDEIELNFGLEATQWGGLDIQ.DGDVAENGTIERNKNANSETPKE
12 TPLGINSSE...TNRYRIIIEFKDS..........GQPQNNLQGMVAKVAFNFDARQVMAPKYHDQNAPNGNLTGNGIIG..DRATTDSTSNVANQPAVVDAPAWQD
13 LFDTVTFANIIEGQ.LDTQQIDMPVRAYAIQ.ATNTGDDKTTVLEQATAAYQKYVNQ..NKGQAGGVTK
14 QWTGLPVNPA.DFDTVEMSIEFVEYSIRDA...RGVEEQNKYQGDTADITFTLEARQWEGQDVS.....DEEGYVESNEQARNGQ
15 TPDGIKAGAGVDDHRFRMMVKFPET..........NVPQNDLQGKQAHLTFNFDARQGAG.TVNNQN......IGN.................ANPATIANPITNAN
16 SPDGIDAGS...YGRYRIMISFPET..........NVAQNKLQGMVAKVDFKLDARQVMGNKSHNAQGPNGTITGNGKQGLTDKTVTPNGTGDLVDPDTTDKAAWEDTNQ
17 IFPPILKAGDHKDWGLIIEGKFADD............ADNHYQGSSCTFNMRCRGSQNSPTEGFEEITGNGAD
18 LFDSITVANVVEDQTLEQTTQNIQVEAFGIQ.ANDIGADDKT...APSDVWKVLVNQGPSKDKTGEDTKTDIVGG
19 EGG..ALESG.ANMSIDIEISFVNNVKKYPN..SLFMEQNKFQGESINLDLIFEATQMSGEDRS
20 EKGGLKAGN...SDTLSVQFEFVEN..........DKDQNEFQGDSLELKWTFDAQQTKG.EKK
21 EIGGLKKGD...KDKLFVQFEFVDN..........GEDQNQFQGDSLALKWTFEGTQPVGKERKN
22 EGDGLEAGT...SDQMYVGFEFVDN..........GEDQNEFQGDALELTWTFEAHQTEG.ELR
23 LLESVTMNPRAQATVTHTKLVSDYDKENKKDRYTFSYDTNSFGYDSAEYRLNITAKTVQATKDAVDEVLTGNREMVPENFDKLAASLKEMCR
24 AGFKYPLGPD...ESFDVQFDVYLN..........KDAGNEYQGSSLTGTLNVEGGQSVDGAEFEDGTETDGSEG
25 EYDGVPKTPA.DYDQVRMEIQFKNDTAKTA...DGLSVQNKFQGNAISLQFSFEATQWNGLTIT.KDHTDKDGYVKENEKAHSEDKN
26 EPDGLKAGT...SDTLSVMFEFVEN..........KKDQNVFQGDSLKLEWTFDAKQTSGSAR
27 EYDGIPKNPF.DYDKMEMIIEFVNDQTKDK...AGHYIQNKYQGDAIQIDLSFEATQWNGLTINPKKHTDEKGYVKENEKAHSEDKK
28 ETVDAGELGGGESRSYTVHFSVPDS............AGNEIQSDKVTLEITADLEQNETQAD
29 KEEGVQNTTSNFLDHLTLSPEVSND...........EHAADYSGCKFIVDFHAEAVPADELAIEVADWDLIQTDAGGLLDWAKLLPES
30 PDRGIPLDPQ.DSDDVFIQITFKDDQTKLG...NGEYAQNKYMNASVDFYFDLEATQWDGVHVD...SDDKNGAINNEQANTPDPVTDGSGGTHSNDVTDND
31 LLAPLPVQVG..SQSLNMSLFFRPE............AGNDIQGDILTMSVTSTLNQDASQ
```

Fig. S1. Sequence alignment of *Bacillus subtilis* TasA, *Bacillus cereus* CalY1/CalY2, and close primary sequences from organisms with varying evolutionary distance generated by ClustalW, covering *Bacillicae*, *Chlostridia*, *Haloferacaceae*, and *Archea*. Residues conserved in more than 90% of the sequences are colored according to the Taylor coloring scheme provided by strap (https://www.bioinformatics.org/strap/aa/) (1). A precise description of the alignment workflow can be found in the Material and Methods section. The conserved last alanine of the signal peptide (residue 27 in TasA) is shown in bold.

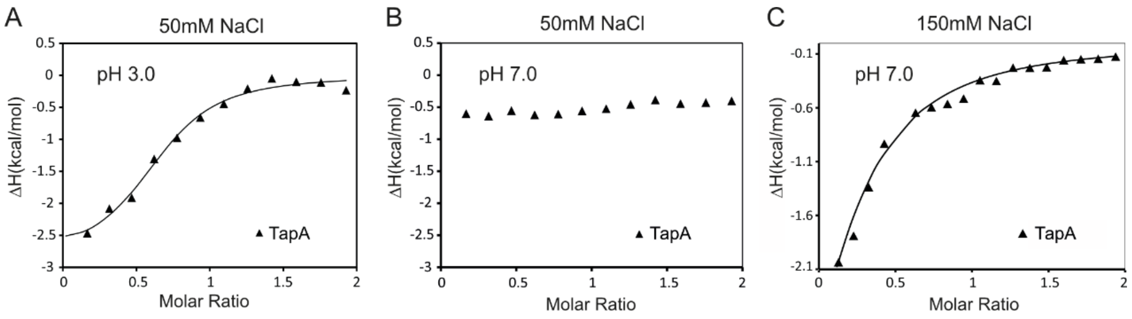

Fig. S2. ITC titration of TapA to TasA in 20 mM Tris HCl buffer with (**A**) 50 mM NaCl at pH 3.0, (**B**) 50 mM NaCl at pH 7.0 and (**C**) 150 mM NaCl at pH 7.0. At physiological pH the interaction necessitates a higher salt concentration. At acidic pH the proteins interact with lower salt but form a different, Thioflavin T stainable, fibril (as shown in SI Appendix, Fig. S3).

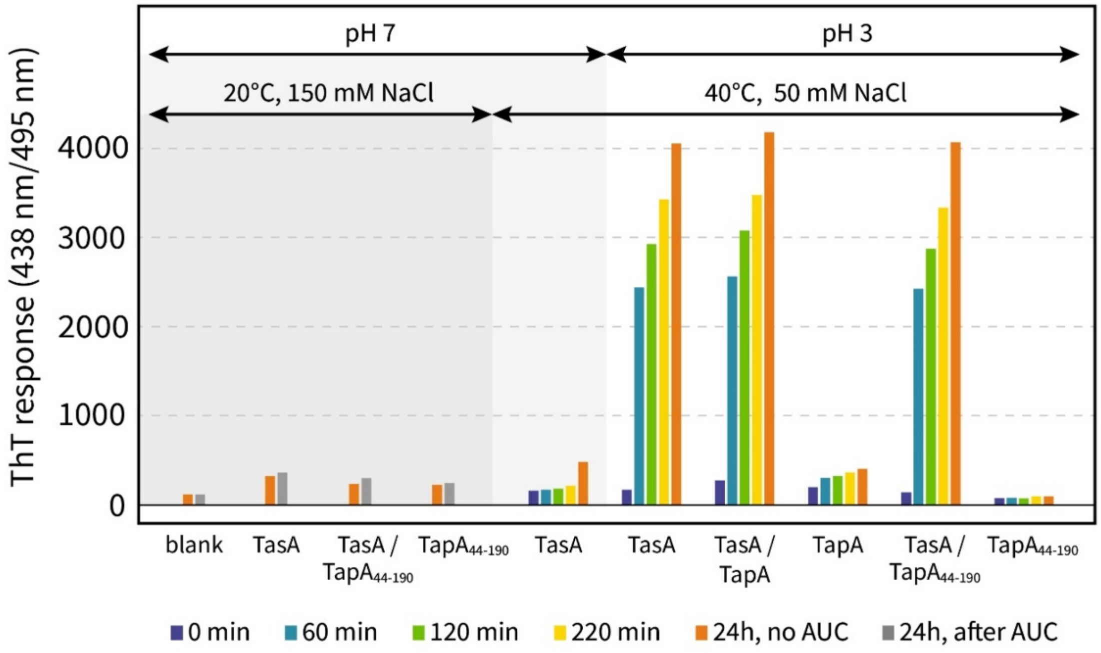

Fig. S3. Thioflavin T (ThT) assay probing stainable fiber formation upon varying multiple factors (pH, temperature, and salt concentration). For ThT response measurements, samples of 10 µl were drawn, diluted with 90 µl of the appropriate buffer and mixed with 100 µl 40 µM pH 7 buffered ThT.

At a sample condition of pH 7 at 20 °C in 20 mM phosphate buffer with 150 mM NaCl, incubation was performed with 100 µM mature $TasA_{28\text{-}261}$, $TapA_{44\text{-}190}$ and a mixture of both (each 100 µM) for 24 h. Under these conditions, TasA/TapA interaction was shown by ITC (SI Appendix, Fig. S2). Little to no signal is observed in the ThT assay under these conditions, indicative of no amyloid fiber species in the sample. Subsequent ultracentrifugation (as performed for filament collection) does not yield a significantly higher ThT response ensuring fiber-free samples for further experiments.

Increasing the temperature to 40 °C and decreasing the salt concentration to 50 mM in 20 mM HEPES causes a minor rise in signal for TasA (see the orange bar after 24 h) while still showing little absolute ThT response.

However, at pH 3 mature $TasA_{28\text{-}261}$ and TapA at 50 µM (each 50 µM when mixed) a different result is observed. Incubation at 40 °C in 20 mM HEPES and 50 mM NaCl shows a strong ThT response for TasA and TasA/TapA mixtures starting with the first datapoint measured after incubation (light blue bar, 60 min). For TapA alone, no significant response is observed. The same tendency remains when truncating the TapA C-terminus ($TapA_{44\text{-}190}$) with TapA itself showing a reduced signal.

Notably, the ThT response for mixtures of TasA and TapA is always smaller than the sum of the individual bars despite the total protein amount being doubled. This speaks toward a fold-protecting effect of TapA that prevents formation of ThT stainable, and thus likely amyloid, fibers when starting from folded protein. This effect is especially pronounced at pH 7, 20 °C and 150 mM NaCl, here shown for $TapA_{44\text{-}190}$.

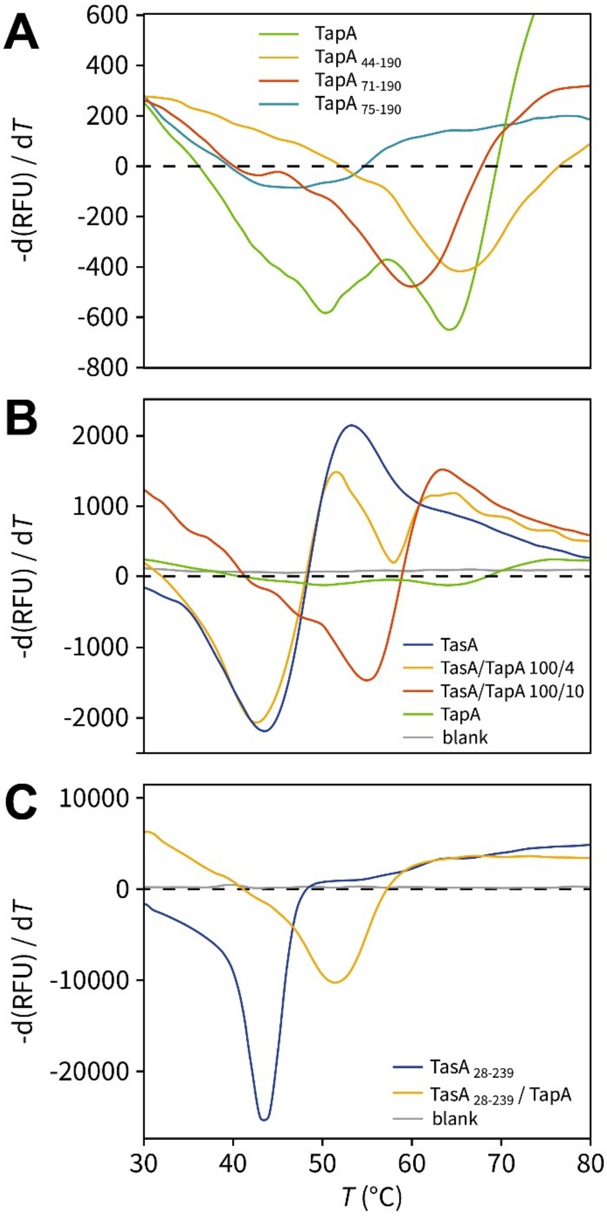

Fig. S4. Thermal shift assays (TSA) probing stability of different TapA variants and the interaction with TasA. All experiments were performed with mature $TapA_{44-253}$ and $TasA_{28-261}$ unless stated otherwise. First derivative calculations of the observed intensity show the melting curve of the individually measured samples.

(**A**) The highest stability is observed for $TapA_{44-190}$ with a T_m of 66 °C. In comparison, $TapA_{71-190}$ is slightly less stable with a T_m of 60 °C, pinpointing a minor contribution of N-terminal residues 44-71. A further truncation of 3 residues at the N-terminus, resulting in $TapA_{75-190}$, shows a significantly lower stability with a T_m of 46°C. Using the mature TapA, a biphasic melting profile with two peaks is observed with one at 64 °C (which likely reflects the melting of the folded domain) and one at 50 °C indicative for the C-terminus losing its association to the folded domain. Interestingly, in our X-Ray studies the most stable construct $TapA_{44-190}$ failed to crystallize in contrast to $TapA_{71-190}$ and $TapA_{75-190}$ despite the latter missing the aromatic ring packing of W74 against Y170 and P171 (Fig. 3G).

(**B**) TapA influences stability of TasA filaments for different mixture ratios, measured after 17 h at 20 °C. Pure TasA is shown in blue with a T_m of 43 °C. With addition of 1/10 of TapA to TasA

an T_m increase by almost 12 °C is detected (red curve). A ratio of 1/25 TapA to TasA is not sufficient to affect the TasA profile (yellow curve).
(**C**) C-terminally truncated $TasA_{28-239}$ (250 µM) shows a melting temperature of 44 °C. Addition of 50 µM TapA increased T_m to 53 °C showing a less pronounced effect than for the mature protein shown in **B**. A blank buffer measurement is shown in gray.

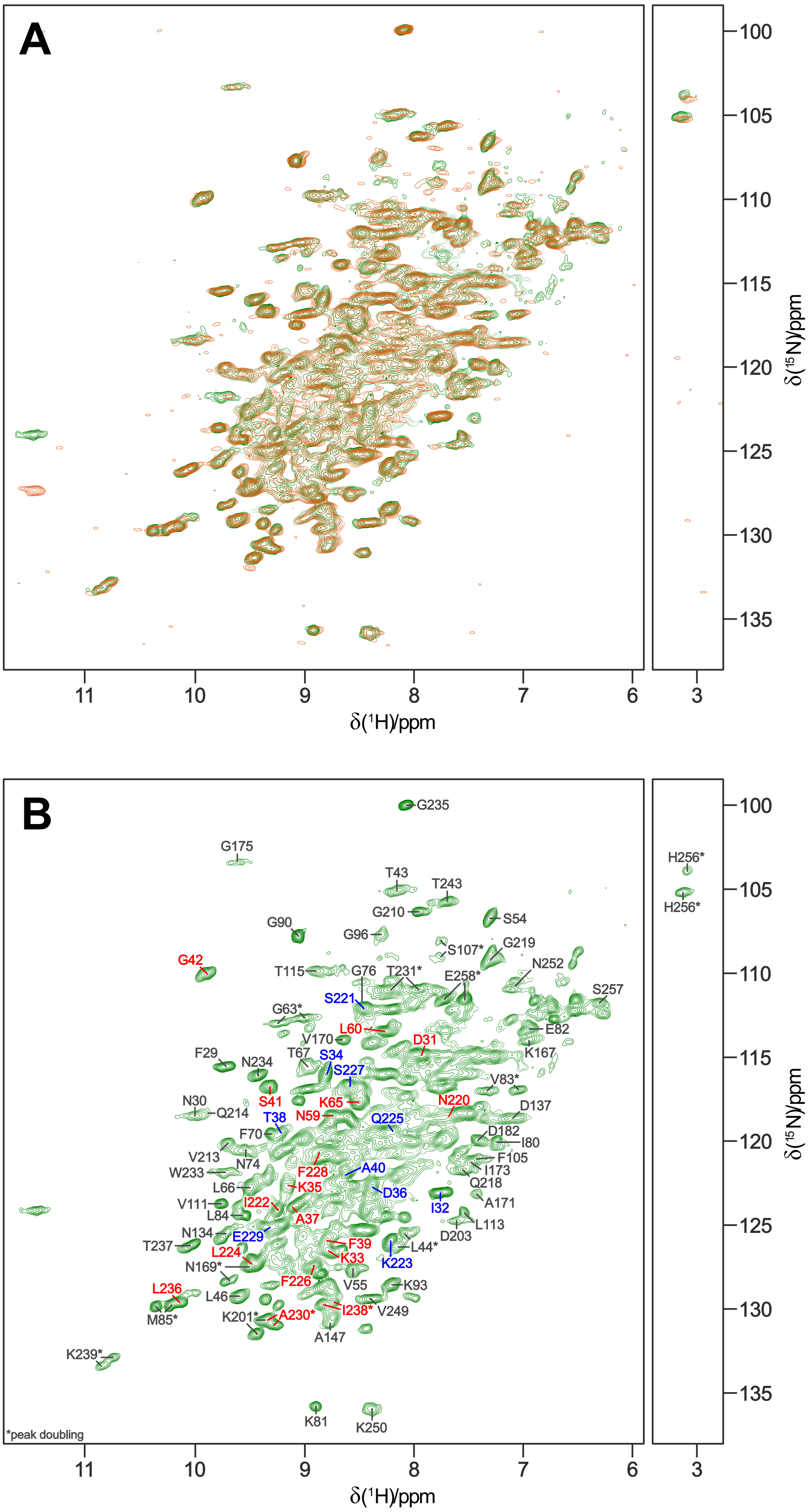
A
100
105
110
115
120
125
130
135
11
10
9
8
7
6
3
δ(¹H)/ppm
δ(¹⁵N)/ppm
B
G235
G175
T43
T243
G210
S54
G90
G96
S107*
G219
G42
N252
T115
G76
T231*
E258*
S257
S221
G63*
L60
E82
V170
D31
F29
T67
S34
K167
N234
S227
V83*
N30
S41
K65
N220
D137
Q214
T38
N59
Q225
F70
D182
I80
V213
N74
F105
W233
F228
A40
I173
L66
K35
Q218
D36
V111
I222
I32
A171
L84
A37
L113
N134
E229
D203
T237
F39
L44*
L224
K33
K223
N169*
F226
V55
K93
L236
L46
M85*
I238*
V249
K201*
A230*
A147
K239*
H256*
H256*
K81
K250
*peak doubling
11
10
9
8
7
6
3
δ(¹H)/ppm
100
105
110
115
120
125
130
135
δ(¹⁵N)/ppm

Fig. S5. (**A**) Comparison of ^1H-^{15}N spectra from TapA-induced filaments of ^2H,^{13}C,^{15}N-labeled (100% back-exchanged) TasA (green) collected by ultracentrifugation with isotopically mixed (^2H,^{15}N/[2-^{13}C]; 50/50) TasA filaments generated at pH 7 through concentration (orange). The histidine sidechain signal at ~11.5 ppm ^1H chemical shift is aliased, its position varies due to differing spectral widths in the ^{15}N dimension. (**B**) Solid-state NMR ^1H-^{15}N correlation of TapA-fibrillated TasA filaments as shown in **A** with assignments indicated for resolved signals. Asterisks indicate residues with peak doubling. Residues that give rise to intense signals presented in SI Appendix, Fig. S8B (antiparallel inter-strand contacts from β0 and β9) and SI Appendix, Fig. S8C (parallel inter-strand contacts) are indicated in red. Further signals arising from β0 (D31-S41) and β9 (A230-N220) are highlighted in blue. For β0 and β9 see also SI Appendix, Fig. S6 which includes strip plots used for assignment. A complete list of assigned chemical shifts can be found in SI Appendix, Tab. S2.

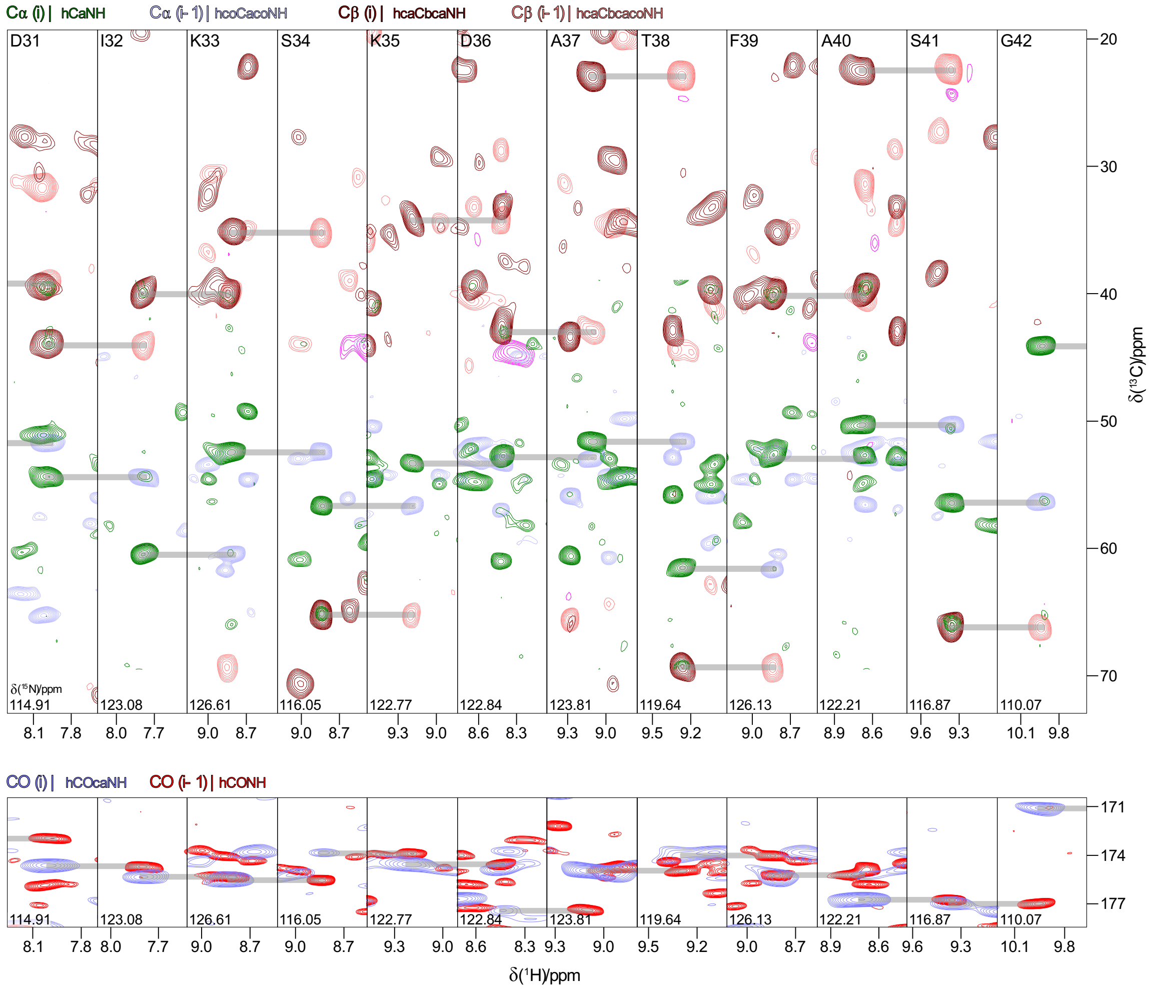

Fig. S6. Strip plots displaying NMR assignment spectra for stretches 31-42 and 220-231 (next page). Spectra are shown in green (hCaNH), light blue (hcoCAcoNH), brown (hcaCbcaNH) and light orange/pink (positive/negative contours of hcaCbcacoNH) for aliphatic carbons on the top and in light blue (hCOcaNH) and red (hCONH) for carbonyls on the bottom. The spectra description above the strip plots denote whether atoms from the amino acid itself (i) or the preceding one (i-1) are measured. Connecting sequential links are highlighted with grey bars. Doubling of peaks is observed for asterisk marked residues 229, 230 and 231 (next page) wheras 231 shows clearly separated peaks in the ^{1}H and ^{13}C dimensions. For the other residues the effect is less pronounced and manifests as line broadening in ^{1}H and ^{13}C for 230 and in ^{1}H for 229.

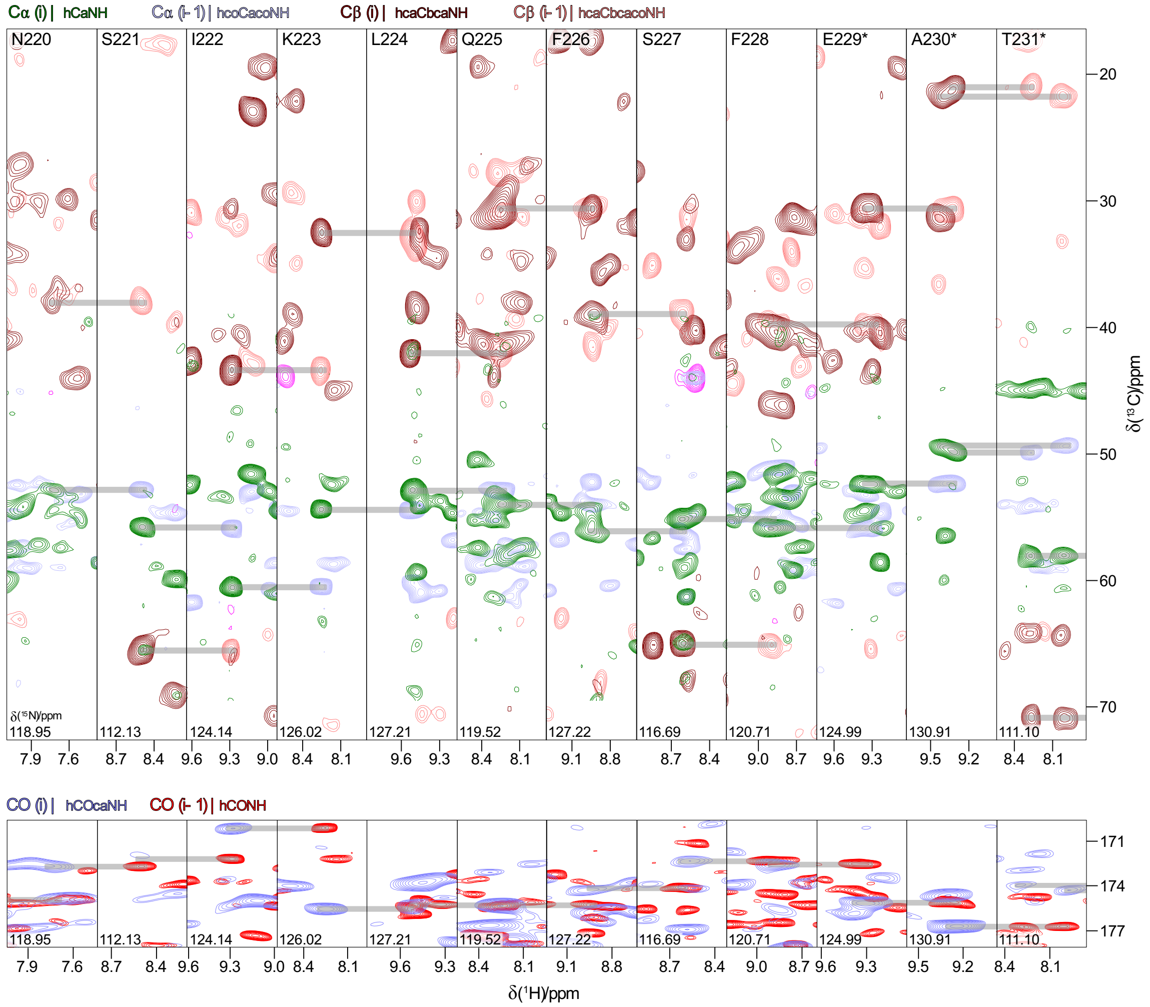

Fig. S6. continued

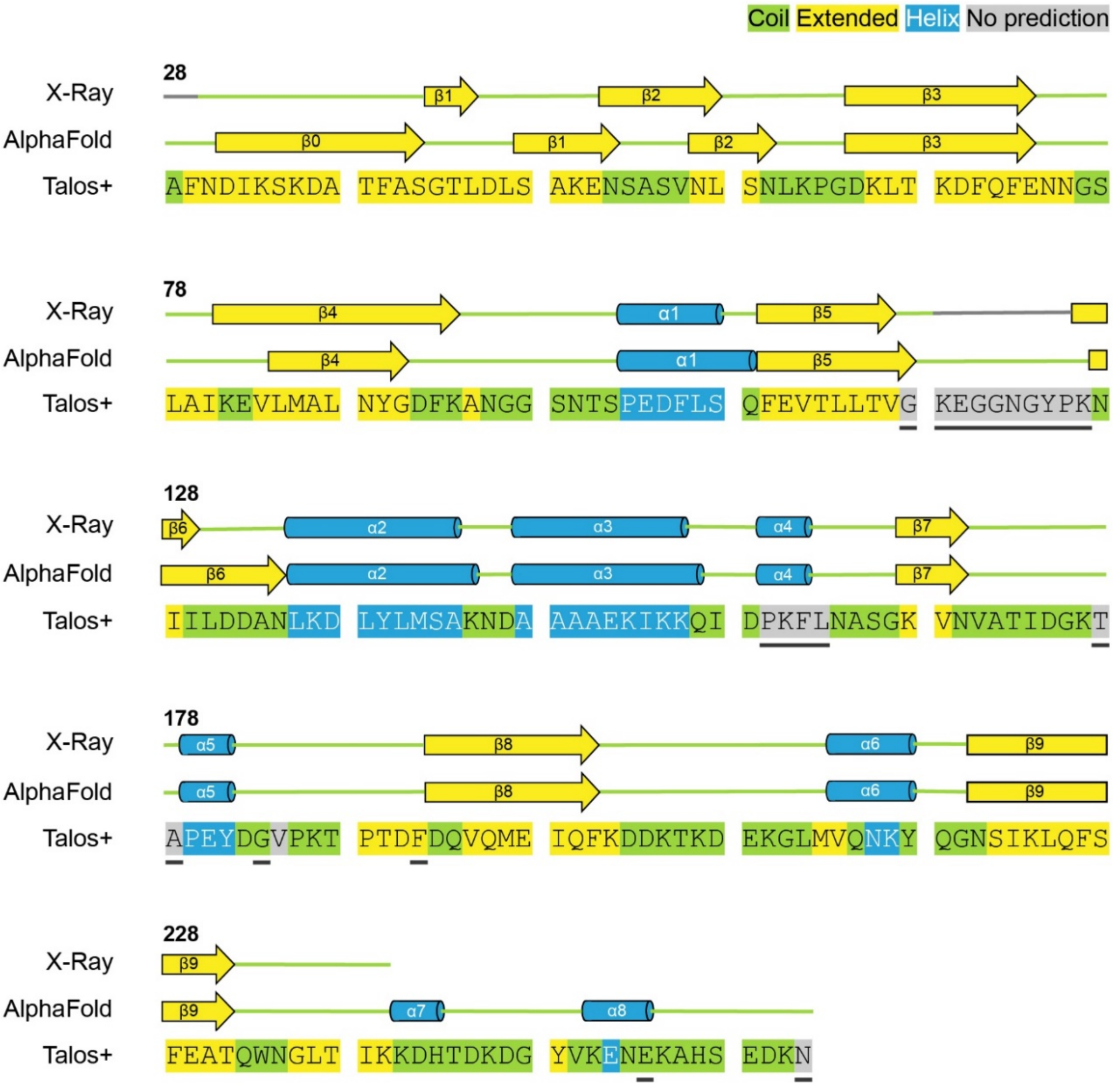

Fig. S7. Comparison of secondary structure information obtained from the X-Ray structure of monomeric TasA (PDB 5OF1), filamentous TasA predicted from AlphaFold multimer and the TALOS+ prediction based on assigned chemical shifts (SI Appendix, Tab. S2). Sheets are colored in yellow, helices in blue, coiled regions in green and not predicted (NMR) or missing regions (X-Ray) in gray. Residues lacking NMR assignments are underlined. For P185 only the Cα shift is known. Fig. 3B in the main text visualized the TALOS+ prediction plotted on the X-Ray structure, SI Appendix, Fig. S11 does the same for the AlphaFold multimer model. Noteworthy is that the stretch between β0 and β1 is present in an extended conformation in the AlphaFold prediction and β6 manifests as an irregular β-sheet in X-Ray and AlphaFold.

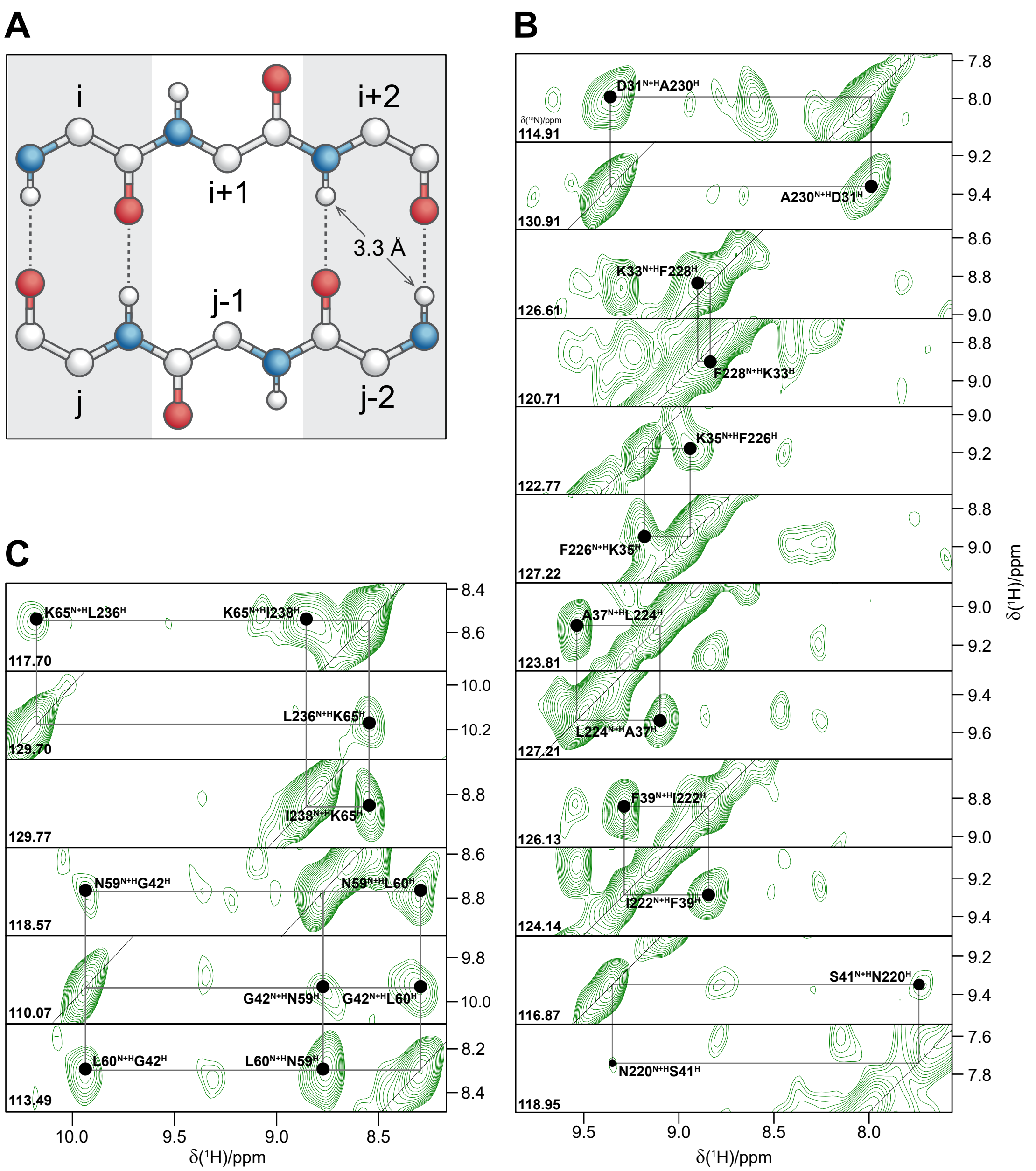

Fig. S8. NMR contacts that can be unambiguously assigned due to typical secondary structure patterns. (**A**) Schematic pattern of an antiparallel β-sheet with the cross-strand H^N-H^N distance of 3.3 Å indicated. (**B**) Slices from the solid-state NMR 3D hNHH spectrum recorded on TapA-fibrillated ^2H,^{13}C,^{15}N-labeled and 100% back-exchanged TasA filaments showing H^N-H^N contacts. Symmetric cross-peaks are characteristic for the antiparallel strands and can be attributed to β0 and β9 (Fig. 3D and 4C, left). (**C**) Additional slices from the spectrum shown in **B**. Contacts between K65 and L236/I238 (Fig. 4C, right), part of a short parallel β-sheet, are displayed on the top. On the bottom, residues G42, N59 and L60 are shown that form a less regular interaction (4C, left).

The peak doubling previously observed for A230 (SI Appendix, Figs. S5B and S6) and I238 (SI Appendix, Fig. S5B) is not resolved and appears as an increase in peak linewidth.

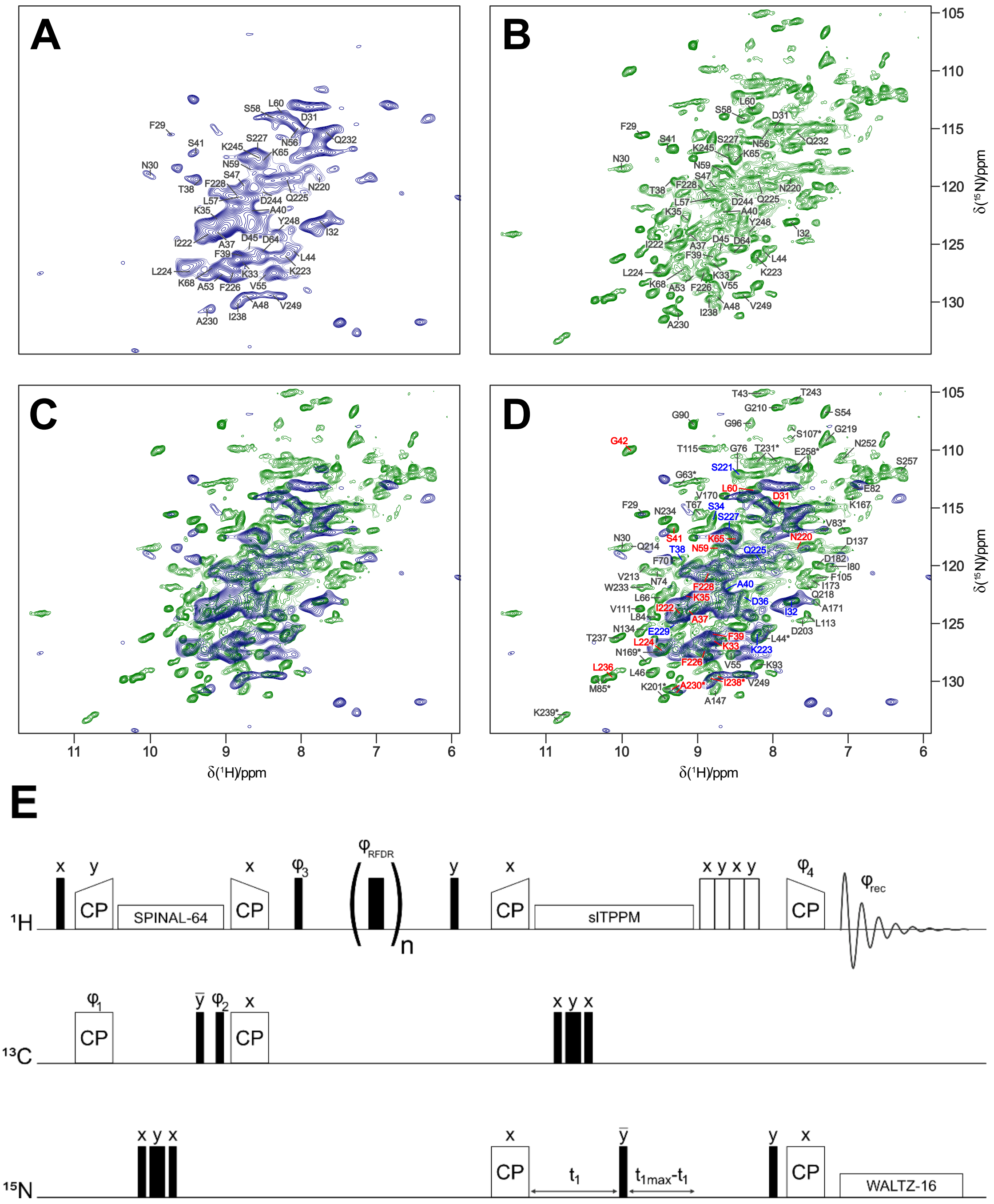

Fig. S9. Intermolecular contacts of TasA filaments monitored by NMR. (**A**) NH plane of a hchhNH experiment acquired on a sample in aqueous buffer prepared with a mixed labeling scheme (^{2}H,^{15}N/[2-^{13}C]; 50/50). Detectable peaks present at the interface are labeled. (**B**) hNH spectrum shown in SI Appendix, Fig. S5 with peak labeling as presented in **A**. (**C**) Superposition of **A** and **B** without labels. (**D**) Superposition of **A** and **B** with peak labeling as presented in SI Appendix, Fig. S5B. (**E**) Pulse sequence used to acquire the spectrum shown in **A**. Initial ^{1}H polarization is produced by a $\pi/2$ pulse. Afterwards, it is transferred to ^{13}C nuclei and back using a ^{1}H/^{13}C CP block which functions as a filter for non ^{13}C labeled material. The magnetization, now at ^{1}H again, is transferred through space to adjacent ^{1}H nuclei using an rotor-synchronized 3 ms RFDR (2) pulse train (n=180 at 60 kHz MAS). Subsequently a second CP block is applied, this time on ^{1}H/^{15}N filtering everything lacking ^{15}N isotopes. At the end of the pulse sequence, only magnetization that was transferred through space and experienced both ^{13}C and ^{15}N remains. For our labelling pattern this is solely true for inter-molecular cross peaks. Black rectangles depict hard pulses (narrow: $\pi/2$; broad: π), empty rectangles are used for decoupling sequences (either SPINAL-64, slTPPM or WALTZ-16), cross-polarization (CP) pulses and the

MISSISSIPPI solvent suppression scheme (xyxy pulse train prior to the last CP). Phase cycling is applied for pulses labeled with φ. φ_1=00002222, φ_2=13, φ_3=3311, φ_4=00001111, φ_{RFDR}=01011010 (cycled within the RFDR sequence), φ_{rec}=20021331**.**

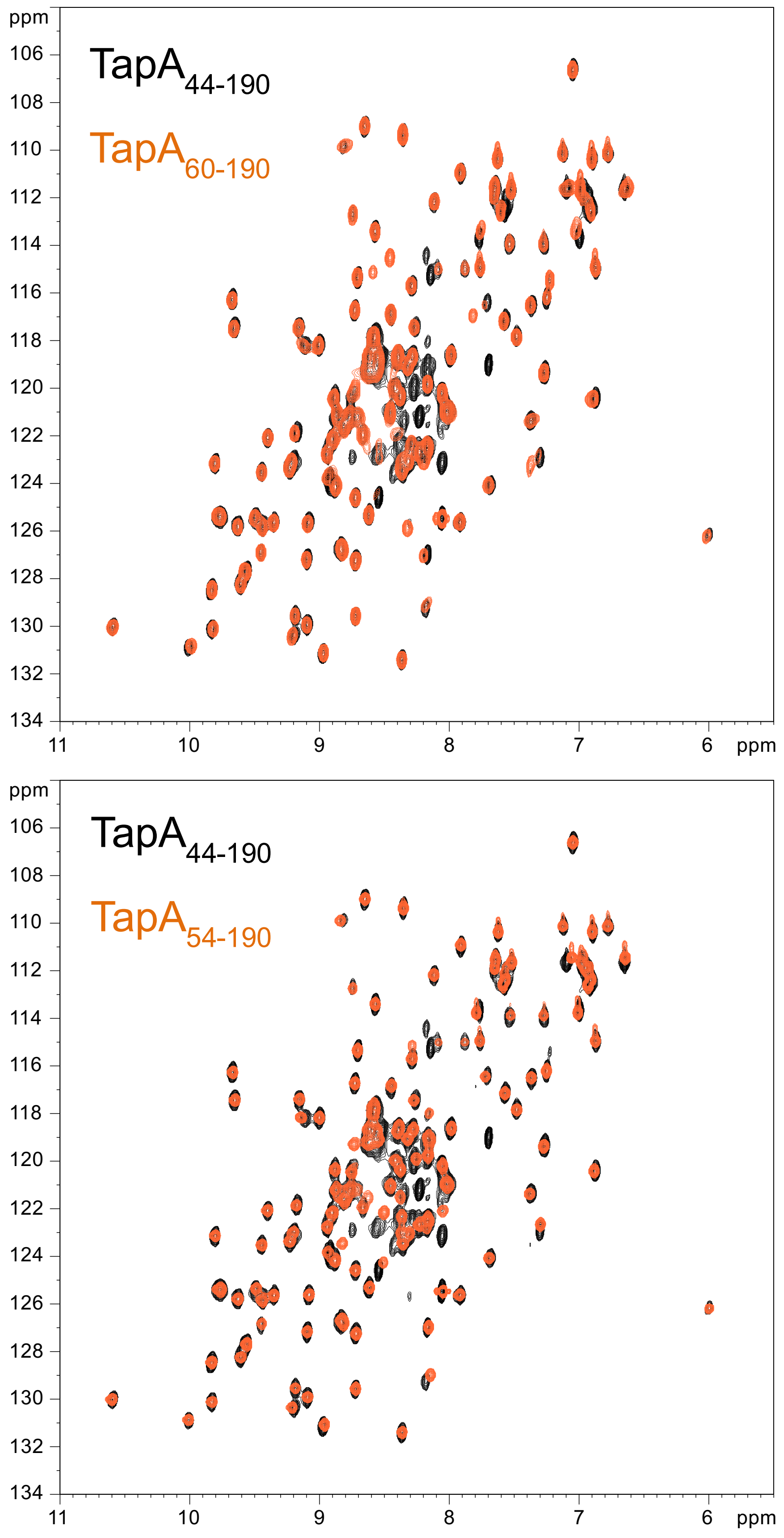

Fig. S10. Overlay of 2D solution NMR ^{1}H-^{15}N correlation spectra of $TapA_{44-190}$ (black) with $TapA_{60-190}$ (orange, top) and $TapA_{54-190}$ (orange, bottom). Black signals without orange overlap are only present in spectral regions characteristic of disordered stretches and indicate an unfolded N-terminus.

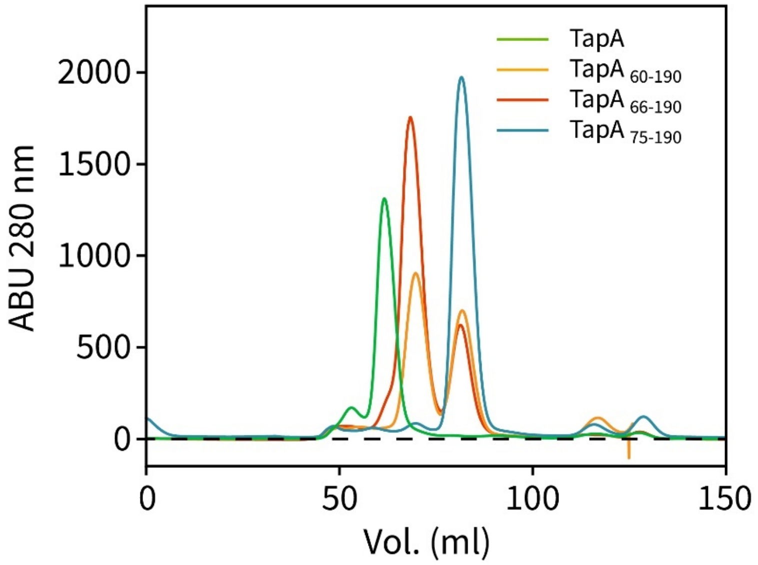

Fig. S11. TapA variants monitored by gel filtration on a Superdex 75 column (total volume 120 ml, buffer: 20 mM phosphate pH 7.0, 150 mM NaCl). Mature TapA (green curve) shows one elution maxima, characteristic of a defined monomer. For TapA variants with three cysteines like $TapA_{60-190}$ (orange curve) and $TapA_{66-190}$ (red curve) two maxima occur indicating an additional species, likely a dimer. As this effect is not present in the presence of reducing agents it can be attributed to the unbound C68 forming a disulfide bridge between two monomers. Truncating more N-terminal residues and removing C68 ($TapA_{75-190}$, blue curve) yields a monomeric profile again.
Taken together it can be concluded that C68 is likely being bound to C58 in the mature protein forming a second disulfide bridge in addition to the one observed in the crystal structure (Fig. 3E, C92-C188).

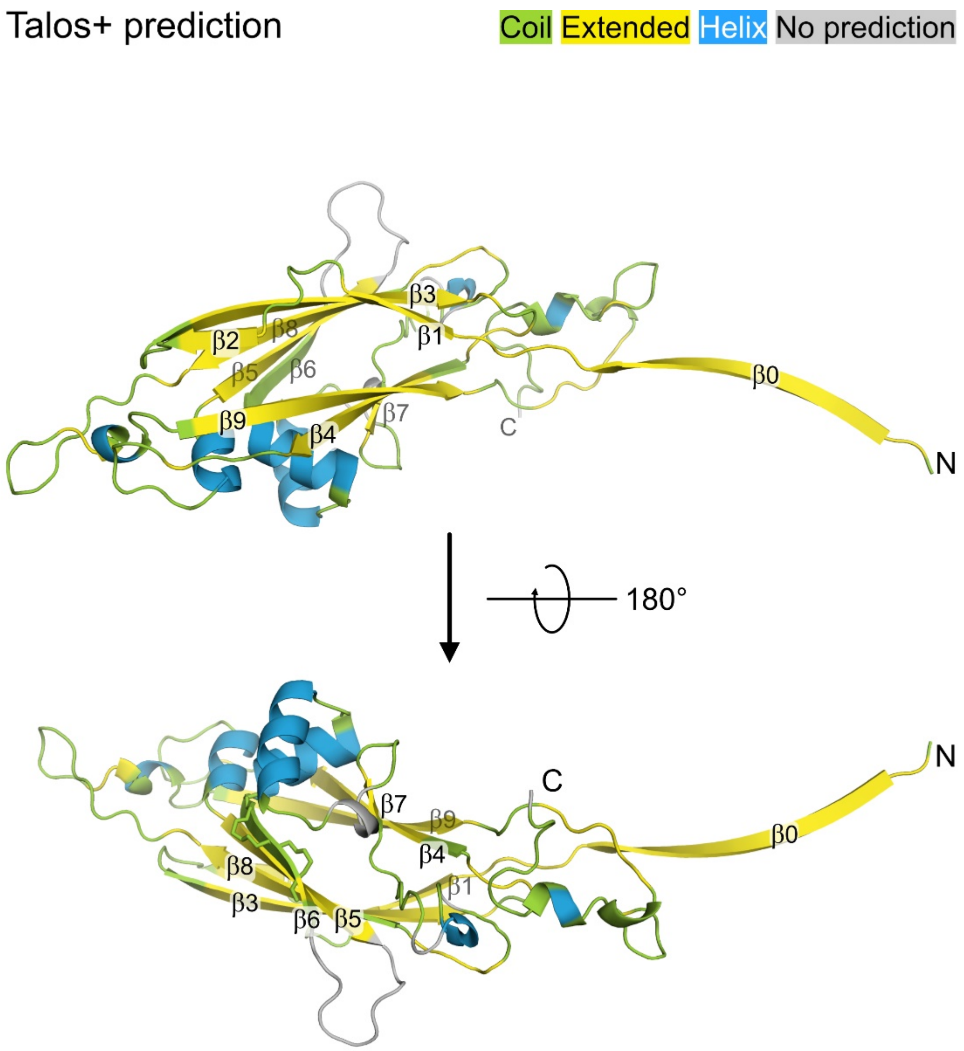

Fig. S12. TALOS+ secondary structure prediction based on solid-state NMR chemical shifts displayed on a cartoon representation of a protomer extracted from an AlphaFold multimer filament (SI Appendix, Fig. S7 and Tab. S2). Predicted helices are shown in blue, sheets in yellow, and loops in green. Regions lacking a prediction are indicated in gray. β6 exists in an irregular conformation, similar to what is observed in the X-Ray structure (PDB 5OF1) and which is represented by the backbone trace shown on the bottom panel.

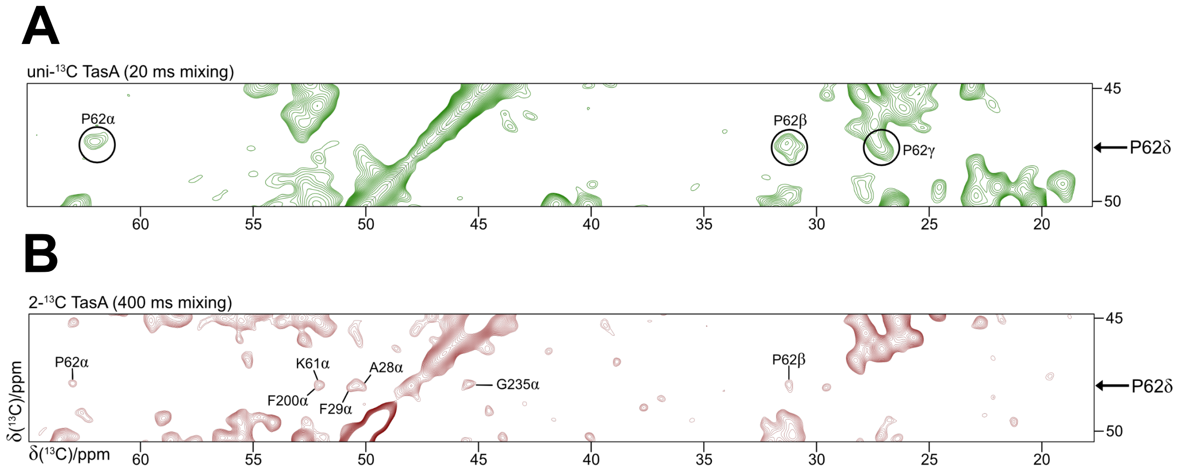

Fig. S13. ^{13}C-^{13}C DARR spectra showing separated peaks of P62. (**A**) Spectrum of uniformly ^{13}C, ^{15}N-labeled TasA fibers at a short mixing time of 20 ms showing intra-residue crosspeaks of P62. (**B**) Spectrum of sparsely labeled TasA fibers composed of [2-^{13}C]-glycerol and uniformly ^{15}N-labeled protein recorded with 400 ms mixing time. P62 shows crosspeaks to A28, F29, K61, F200 and G235 consistent with the predicted interface area shown in Fig. 4E. Assignment of crosspeaks was conducted by applying a correction (3) to the chemical shifts observed for the perdeuterated protein (SI Appendix, Tab. S2).

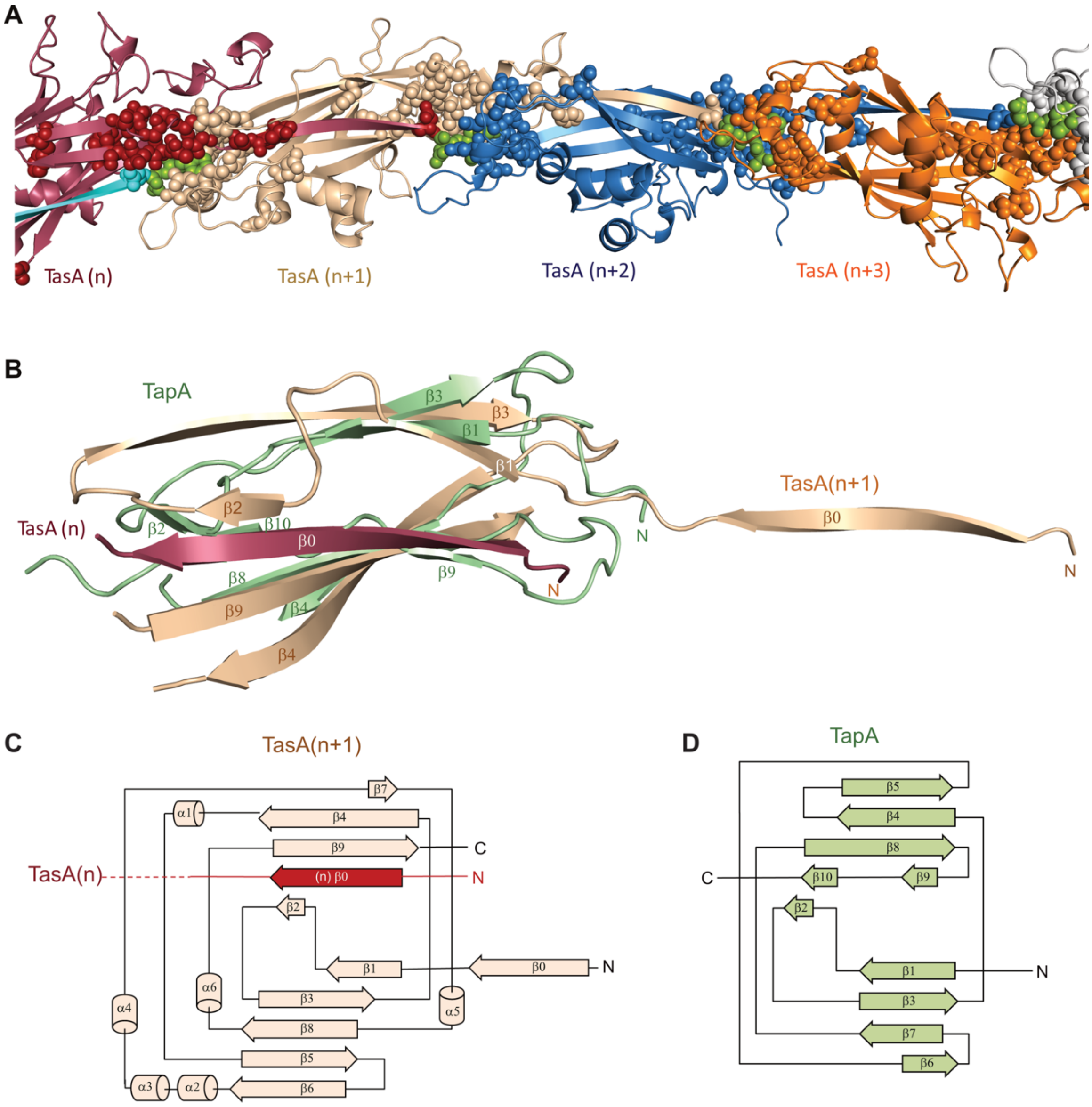

Fig. S14. (**A**) AlphaFold predicted TasA filament chain with the side chains of the most conserved residues indicated as spheres (see SI Appendix, Figs. S1 and Fig. 4A). All phenylalanine and alanine residues at the N-termini (A28, F29) are shown in green, other residues in the color of their protomer. (**B**) Overlay of TapA (green) with filamentous TasA (beige and dark red). Secondary structure topology plots of filamentous TasA (**C**) and TapA (**D**) with the same colours as in **B**.

Table S1. Results of AUC measurements: *s*-values, frictional ratios and apparent molecular weights.

Protein sample	Conc. [µM]	f/f_0	Monomer $S_{20,w}$	Monomer MW [kDa]	Oligomer[a,b,e] $S_{20,w}$	Oligomer[a,b,e] MW [kDa]
$TapA_{44\text{-}62\ mutant}$[d]	85	1.28	0.60	2.8	-	-
$TapA_{75\text{-}190}$	100	1.24	1.69	14.9	-	-
$TapA_{44\text{-}190}$	100	1.33	1.81	17.5	-	-
$TapA_{44\text{-}253}$	100	1.74	1.90	27.0	-	-
$TasA_{28\text{-}261}$	100	1.45	2.15	25.4	-	-
$TasA_{28\text{-}261}$[c]	100	1.35	2.97	37.2	-	-
$TasA_{28\text{-}261}$+$TapA_{44\text{-}62\ mutant}$[d]	100 + 250	1.69	2.15	32.1	9.7	~ 300
$TasA_{28\text{-}261}$ + $TapA_{75\text{-}190}$	100 + 100	1.37	1.93	20.5	-	-
$TasA_{28\text{-}261}$ + $TapA_{44\text{-}190}$	100 + 100	1.67	1.83	24.9	6.7	~ 170
$TasA_{28\text{-}261}$ + $TapA_{44\text{-}253}$	100 + 1	1.54	2.17	28.3	-	-
$TasA_{28\text{-}261}$ + $TapA_{44\text{-}253}$	100 + 2	1.58	2.18	29.4	9.8	~ 280
$TasA_{28\text{-}261}$ + $TapA_{44\text{-}253}$	100 + 4	1.81	2.05	33.0	9.6	~ 330
$TasA_{28\text{-}261}$ + $TapA_{44\text{-}253}$	100 + 10	1.95	1.94	34.3	8.8	~ 330
$TasA_{28\text{-}261}$ + $TapA_{44\text{-}253}$	100 + 70	1.98	1.87	32.3	6.0	~ 190
$TasA_{28\text{-}261}$[c] + $TapA_{44\text{-}253}$	100 + 100	2.17	1.83	36.8	10.5	~ 500

[a] Largest oligomeric species formed over the course of the sedimentation velocity experiment.

[b] Potential protein fibrils could not be detected due to the high rotor speed of 40,000 rpm

[c] Experiment performed with isotopic labeled ^{2}H,^{13}C,^{15}N-$TasA_{28\text{-}261}$

[d] $TapA_{44\text{-}62\ wt}$: AFHDIETFDVSLQTCKDFQ; $TapA_{44\text{-}62\ mutant}$: AFHDIETFDVSLQTSKDAQ.

[e] Heterogeneous samples such as TapA-induced TasA filaments only allow to determine an approximate MW.

Table S2. Backbone assignments obtained from solid-state NMR of TasA filaments. Signal sets arising form doubled peaks are shown in italics.
Determined chemical shifts, peak lists and raw datasets have been deposited in the BMRB with the accession code 51785. Processed spectra and the CCPN 2.4.2 assignment project are uploaded to zenodo (10.5281/zenodo.7534572).

Res.	Nuc.	Shift	Res.	Nuc.	Shift	Res.	Nuc.	Shift	Res.	Nuc.	Shift
A28	C	172.04	I80	CB	38.76	*A143*	*CA*	*52.93*	E208	HN	9.01
A28	CA	50.20	I80	HN	7.22	*A143*	*CB*	*19.49*	E208	N	116.90
A28	CB	19.53	I80	N	120.18	*A143*	*HN*	*9.00*	K209	C	176.61
F29	C	176.38	K81	C	173.48	*A143*	*N*	*124.21*	K209	CA	55.27
F29	CA	50.80	K81	CA	59.09	K144	C	176.35	K209	CB	31.38
F29	CB	39.77	K81	CB	30.32	K144	CA	53.64	K209	HN	8.28
F29	HN	9.76	K81	HN	8.93	K144	CB	31.55	K209	N	119.44
F29	N	115.61	K81	N	135.94	K144	HN	6.55	G210	C	174.22
N30	C	172.98	E82	C	173.94	K144	N	112.25	G210	CA	44.35
N30	CA	51.81	E82	CA	53.36	N145	C	174.84	G210	HN	7.98
N30	CB	39.28	E82	CB	31.74	N145	CA	53.14	G210	N	106.36
N30	HN	10.04	E82	HN	6.99	N145	CB	36.99	L211	C	177.26
N30	N	118.55	E82	N	112.92	N145	HN	7.73	L211	CA	53.22
D31	C	174.68	V83	C	173.69	N145	N	119.52	L211	CB	40.80
D31	CA	54.39	V83	CA	61.63	D146	C	176.45	L211	HN	8.39
D31	CB	43.97	V83	CB	31.00	D146	CA	54.65	L211	N	123.77
D31	HN	7.98	V83	HN	7.37	D146	CB	44.06	M212	C	175.36
D31	N	114.89	V83	N	117.06	D146	HN	7.53	M212	CA	53.30
I32	C	175.36	*V83*	*C*	*173.60*	D146	N	118.43	M212	CB	33.02
I32	CA	60.48	*V83*	*CA*	*61.75*	A147	C	180.05	V213	C	172.79
I32	CB	39.98	*V83*	*CB*	*31.01*	A147	CA	54.58	V213	CA	58.58
I32	HN	7.79	*V83*	*HN*	*7.07*	A147	CB	17.82	V213	CB	35.12
I32	N	123.08	*V83*	*N*	*116.97*	A147	HN	8.79	V213	HN	9.73
K33	C	175.52	L84	C	176.59	A147	N	130.76	V213	N	120.21
K33	CA	52.43	L84	CA	52.39	A148	C	181.25	Q214	C	176.71
K33	CB	35.21	L84	CB	42.72	A148	CA	54.47	Q214	CA	57.53
K33	HN	8.81	L84	HN	9.60	A148	CB	16.77	Q214	CB	27.66
K33	N	126.61	L84	N	124.23	A148	HN	8.20	Q214	HN	9.82
S34	C	173.90	M85	C	174.37	A148	N	122.19	Q214	N	118.23
S34	CA	56.68	M85	CA	54.62	A149	C	179.60	N215	C	178.40
S34	CB	65.18	M85	CB	34.95	A149	CA	54.46	N215	CA	55.43
S34	HN	8.83	M85	HN	10.39	A149	CB	18.80	N215	CB	37.94
S34	N	116.05	M85	N	129.89	A149	HN	8.75	N215	HN	8.11
K35	C	174.57	*M85*	*C*	*174.38*	A149	N	123.42	N215	N	117.36
K35	CA	53.32	*M85*	*CA*	*54.28*	A150	C	179.51	K216	C	174.77
K35	CB	34.15	*M85*	*CB*	*34.79*	A150	CA	54.46	K216	CA	57.53
K35	HN	9.19	*M85*	*HN*	*10.25*	A150	CB	17.28	K216	CB	29.83
K35	N	122.78	*M85*	*N*	*129.76*	A150	HN	8.03	K216	HN	7.62
D36	C	177.43	A86	C	173.85	A150	N	118.26	K216	N	118.35
D36	CA	52.94	A86	CA	49.32	*A150*	*C*	*179.49*	Y217	C	176.83
D36	CB	42.77	A86	CB	22.14	*A150*	*CA*	*54.30*	Y217	CA	57.08
D36	HN	8.42	A86	HN	8.69	*A150*	*CB*	*17.42*	Y217	CB	37.55
D36	N	122.84	A86	N	126.47	*A150*	*HN*	*8.12*	Y217	HN	7.74
A37	C	174.98	*A86*	*C*	*174.02*	*A150*	*N*	*118.07*	Y217	N	116.12
A37	CA	51.59	*A86*	*CA*	*49.45*	E151	C	178.86	*Y217*	*C*	*176.77*
A37	CB	22.92	*A86*	*CB*	*22.09*	E151	CA	58.62	*Y217*	*CA*	*57.37*

A37	HN	9.11	*A86*	*HN*	*8.45*	E151	CB	28.42	*Y217*	*CB*	*37.51*
A37	N	123.81	*A86*	*N*	*125.36*	E151	HN	7.81	*Y217*	*HN*	*7.55*
T38	C	174.04	L87	C	173.49	E151	N	116.26	*Y217*	*N*	*116.08*
T38	CA	61.62	L87	CA	52.63	K152	C	179.96	Q218	C	176.66
T38	CB	69.30	L87	CB	45.57	K152	CA	58.84	Q218	CA	58.33
T38	HN	9.27	L87	HN	7.74	K152	CB	31.35	Q218	CB	27.60
T38	N	119.64	L87	N	120.02	K152	HN	7.41	Q218	HN	7.56
F39	C	175.12	N88	C	173.13	K152	N	118.18	Q218	N	121.62
F39	CA	52.89	N88	CA	50.40	I153	C	178.72	G219	C	174.94
F39	CB	40.16	N88	CB	41.58	I153	CA	62.18	G219	CA	45.19
F39	HN	8.85	N88	HN	8.35	I153	CB	35.06	G219	HN	7.32
F39	N	126.13	N88	N	120.10	I153	HN	8.09	G219	N	109.25
A40	C	176.77	Y89	C	173.63	I153	N	120.00	N220	C	172.67
A40	CA	50.32	Y89	CA	55.75	K154	C	177.99	N220	CA	52.83
A40	CB	22.45	Y89	CB	41.57	K154	CA	59.05	N220	CB	38.00
A40	HN	8.69	Y89	HN	8.27	K154	CB	31.48	N220	HN	7.74
A40	N	122.22	Y89	N	116.00	K154	HN	8.76	N220	N	118.82
S41	C	177.00	G90	C	171.28	K154	N	119.79	S221	C	172.21
S41	CA	56.42	G90	CA	44.10	K155	C	177.11	S221	CA	55.82
S41	CB	66.18	G90	HN	9.09	K155	CA	57.47	S221	CB	65.55
S41	HN	9.36	G90	N	107.82	K155	CB	31.54	S221	HN	8.49
S41	N	116.79	D91	C	174.31	K155	HN	7.29	S221	N	112.13
G42	C	171.05	D91	CA	54.69	K155	N	114.85	I222	C	170.14
G42	CA	44.11	D91	CB	40.34	Q156	C	174.62	I222	CA	60.55
G42	HN	9.93	D91	HN	8.50	Q156	CA	54.85	I222	CB	43.37
G42	N	110.07	D91	N	117.01	Q156	CB	30.20	I222	HN	9.28
T43	C	172.22	F92	C	175.54	Q156	HN	8.06	I222	N	124.14
T43	CA	58.66	F92	CA	57.69	Q156	N	115.26	K223	C	175.55
T43	CB	71.63	F92	CB	39.50	I157	CA	59.31	K223	CA	54.43
T43	HN	8.20	F92	HN	8.61	I157	CB	40.49	K223	CB	32.50
T43	N	105.17	F92	N	118.65	I157	HN	7.63	K223	HN	8.25
L44	C	173.95	K93	C	173.48	I157	N	120.81	K223	N	126.02
L44	CA	52.44	K93	CA	54.13	D158	CA	53.58	L224	C	175.38
L44	CB	45.01	K93	CB	34.72	D158	HN	9.08	L224	CA	52.90
L44	HN	8.18	K93	HN	8.23	D158	N	119.92	L224	CB	42.04
L44	N	126.30	K93	N	128.59	N163	C	176.13	L224	HN	9.52
L44	*CA*	*52.11*	A94	CA	52.50	N163	CA	51.90	L224	N	127.28
L44	*CB*	*44.79*	A94	CB	18.26	N163	CB	41.74	Q225	C	175.34
L44	*HN*	*8.05*	A94	HN	8.30	A164	C	177.97	Q225	CA	54.01
L44	*N*	*125.49*	A94	N	124.50	A164	CA	54.25	Q225	CB	30.66
D45	C	173.07	N95	C	174.99	A164	CB	17.50	Q225	HN	8.24
D45	CA	53.08	N95	HN	8.78	A164	HN	8.94	Q225	N	119.52
D45	CB	43.29	N95	N	121.42	A164	N	127.46	F226	C	174.15
D45	HN	8.80	G96	C	175.03	S165	C	174.76	F226	CA	56.14
D45	N	125.55	G96	CA	44.90	S165	CA	58.88	F226	CB	38.95
L46	C	175.36	G96	HN	8.33	S165	CB	64.11	F226	HN	8.95
L46	CA	53.06	G96	N	107.64	S165	HN	8.29	F226	N	127.22
L46	CB	44.09	G97	C	173.92	S165	N	111.43	S227	C	172.35
L46	HN	9.62	G97	CA	45.03	G166	C	171.90	S227	CA	55.16
L46	N	129.31	G97	HN	7.84	G166	CA	44.65	S227	CB	65.05
S47	C	173.55	G97	N	111.04	G166	HN	8.18	S227	HN	8.62

**S47**	CA	55.97	**S98**	C	176.37	**G166**	N	110.81	**S227**	N	116.69
**S47**	CB	62.39	**S98**	CA	59.51	**K167**	C	174.67	**F228**	C	172.57
**S47**	HN	8.69	**S98**	CB	62.75	**K167**	CA	55.46	**F228**	CA	55.91
**S47**	N	119.77	**S98**	HN	8.48	**K167**	CB	36.03	**F228**	CB	39.81
S47	*CA*	*56.04*	**S98**	N	115.89	**K167**	HN	6.99	**F228**	HN	8.90
S47	*CB*	*62.96*	**N99**	C	173.88	**K167**	N	113.61	**F228**	N	120.71
S47	*HN*	*8.84*	**N99**	CA	54.83	**V168**	C	175.87	**E229**	C	175.11
S47	*N*	*119.12*	**N99**	CB	39.66	**V168**	CA	60.13	**E229**	CA	52.42
**A48**	C	174.75	**N99**	HN	9.05	**V168**	CB	33.75	**E229**	CB	30.64
**A48**	CA	49.77	**N99**	N	119.89	**V168**	HN	7.72	**E229**	HN	9.33
**A48**	CB	20.08	**T100**	C	174.41	**V168**	N	116.74	**E229**	N	124.99
**A48**	HN	8.80	**T100**	CA	60.02	**N169**	C	173.92	**A230**	C	176.76
**A48**	N	130.00	**T100**	CB	69.03	**N169**	CA	52.70	**A230**	CA	49.87
A48	*C*	*174.75*	**T100**	HN	8.23	**N169**	CB	38.01	**A230**	CB	21.11
A48	*CA*	*49.89*	**T100**	N	112.14	**N169**	HN	9.49	**A230**	HN	9.33
A48	*CB*	*19.87*	**S101**	CA	52.99	**N169**	N	127.30	**A230**	N	130.91
A48	*HN*	*8.77*	**S101**	CB	62.80	***N169***	*C*	*174.16*	***A230***	*C*	*176.74*
A48	*N*	*128.93*	**S101**	HN	7.45	***N169***	*CA*	*53.24*	***A230***	*CA*	*49.42*
**K49**	C	174.76	**S101**	N	115.01	***N169***	*CB*	*38.45*	***A230***	*CB*	*21.67*
**K49**	CA	54.40	**P102**	C	178.59	***N169***	*HN*	*9.73*	***A230***	*HN*	*9.40*
**K49**	CB	34.44	**P102**	CA	63.64	***N169***	*N*	*128.33*	***A230***	*N*	*130.81*
**K49**	HN	8.86	**P102**	CB	31.37	**V170**	C	173.82	**T231**	C	174.31
**K49**	N	123.69	**E103**	C	178.41	**V170**	CA	58.57	**T231**	CA	58.00
K49	*C*	*174.62*	**E103**	CA	60.22	**V170**	CB	30.22	**T231**	CB	70.90
K49	*CA*	*54.41*	**E103**	CB	27.67	**V170**	HN	8.68	**T231**	HN	8.00
K49	*CB*	*34.80*	**E103**	HN	8.17	**V170**	N	114.01	**T231**	N	111.10
K49	*HN*	*8.71*	**E103**	N	114.51	**A171**	C	176.73	***T231***	*C*	*173.98*
K49	*N*	*123.49*	**D104**	C	179.95	**A171**	CA	49.40	***T231***	*CA*	*58.00*
**E50**	C	175.20	**D104**	CA	57.02	**A171**	CB	17.63	***T231***	*CB*	*70.83*
**E50**	CA	54.74	**D104**	CB	38.00	**A171**	HN	7.45	***T231***	*HN*	*8.27*
**E50**	CB	29.51	**D104**	HN	8.08	**A171**	N	123.09	***T231***	*N*	*110.99*
**E50**	HN	8.94	**D104**	N	119.89	**T172**	C	174.83	**Q232**	C	177.11
**E50**	N	123.63	**F105**	C	177.34	**T172**	CA	61.36	**Q232**	CA	53.52
**N51**	C	174.31	**F105**	CA	58.90	**T172**	HN	9.03	**Q232**	CB	32.17
**N51**	CA	51.80	**F105**	CB	37.76	**T172**	N	121.05	**Q232**	HN	7.69
**N51**	CB	38.78	**F105**	HN	7.48	**I173**	C	175.81	**Q232**	N	115.50
**N51**	HN	8.48	**F105**	N	121.13	**I173**	CA	58.28	***Q232***	*C*	*177.15*
**N51**	N	125.46	**L106**	C	177.63	**I173**	CB	34.36	***Q232***	*CA*	*53.53*
**S52**	C	173.18	**L106**	CA	57.45	**I173**	HN	7.50	***Q232***	*CB*	*31.93*
**S52**	CA	56.95	**L106**	CB	40.83	**I173**	N	121.34	***Q232***	*HN*	*7.50*
**S52**	CB	62.79	**L106**	HN	8.37	**D174**	C	177.20	***Q232***	*N*	*114.88*
**S52**	HN	7.32	**L106**	N	118.31	**D174**	CA	55.05	**W233**	C	173.89
**S52**	N	114.88	***L106***	*CA*	*57.41*	**D174**	CB	40.67	**W233**	CA	57.54
**A53**	C	177.90	***L106***	*CB*	*40.85*	**D174**	HN	9.28	**W233**	CB	27.19
**A53**	CA	54.67	***L106***	*HN*	*8.42*	**D174**	N	129.82	**W233**	HN	9.75
**A53**	CB	17.29	***L106***	*N*	*119.43*	**G175**	C	173.36	**W233**	N	121.95
**A53**	HN	9.19	**S107**	C	175.12	**G175**	CA	45.19	**N234**	C	173.29
**A53**	N	127.60	**S107**	CA	58.98	**G175**	HN	9.61	**N234**	CA	52.38
**S54**	C	173.76	**S107**	CB	63.11	**G175**	N	103.43	**N234**	CB	38.22
**S54**	CA	57.07	**S107**	HN	7.82	**K176**	C	177.11	**N234**	HN	9.45
**S54**	CB	64.28	**S107**	N	109.02	**K176**	CA	59.68	**N234**	N	116.10

**S54**	HN	7.34	***S107***	*CA*	*58.84*	**K176**	CB	33.18	**G235**	C	172.42
**S54**	N	106.73	***S107***	*CB*	*63.07*	**K176**	HN	7.80	**G235**	CA	45.24
**V55**	C	175.85	***S107***	*HN*	*7.77*	**K176**	N	120.02	**G235**	HN	8.10
**V55**	CA	65.26	***S107***	*N*	*108.15*	**P179**	C	178.57	**G235**	N	100.02
**V55**	CB	31.70	**Q108**	C	172.55	**P179**	CA	64.35	**L236**	C	175.59
**V55**	HN	8.59	**Q108**	CA	54.03	**P179**	CB	31.12	**L236**	CA	52.47
**V55**	N	127.68	**Q108**	CB	27.06	**E180**	C	177.52	**L236**	CB	45.29
**N56**	C	174.58	**Q108**	HN	8.00	**E180**	CA	57.82	**L236**	HN	10.18
**N56**	CA	51.12	**Q108**	N	118.86	**E180**	CB	30.12	**L236**	N	129.72
**N56**	CB	39.41	***Q108***	*CA*	*54.19*	**E180**	HN	8.66	**T237**	C	174.52
**N56**	HN	8.02	***Q108***	*CB*	*27.14*	**E180**	N	118.52	**T237**	CA	63.05
**N56**	N	114.86	***Q108***	*HN*	*8.08*	***E180***	*CA*	*57.93*	**T237**	CB	66.09
**L57**	C	175.64	***Q108***	*N*	*119.29*	***E180***	*CB*	*30.54*	**T237**	HN	10.08
**L57**	CA	53.36	**F109**	C	174.99	***E180***	*HN*	*8.79*	**T237**	N	126.29
**L57**	CB	46.20	**F109**	CA	55.90	***E180***	*N*	*118.98*	**I238**	C	176.94
**L57**	HN	8.93	**F109**	CB	41.24	**Y181**	C	174.52	**I238**	CA	61.51
**L57**	N	121.14	**F109**	HN	7.68	**Y181**	CA	58.75	**I238**	CB	36.43
L57	*CA*	*53.54*	**F109**	N	116.06	**Y181**	CB	38.85	**I238**	HN	8.86
L57	*CB*	*46.21*	**E110**	C	174.75	**Y181**	HN	8.08	**I238**	N	129.69
L57	*HN*	*8.81*	**E110**	CA	53.30	**Y181**	N	116.49	***I238***	*C*	*176.94*
L57	*N*	*120.76*	**E110**	CB	32.31	**D182**	CA	53.76	***I238***	*CA*	*61.56*
**S58**	C	174.53	**E110**	HN	9.08	**D182**	CB	42.31	***I238***	*CB*	*36.27*
**S58**	CA	55.39	**E110**	N	117.64	**D182**	HN	7.45	***I238***	*HN*	*8.78*
**S58**	CB	64.06	**V111**	C	175.62	**D182**	N	119.94	***I238***	*N*	*129.68*
**S58**	HN	8.41	**V111**	CA	61.23	**V184**	CA	64.95	**K239**	C	177.95
**S58**	N	114.23	**V111**	CB	31.53	**V184**	CB	30.04	**K239**	CA	52.99
**N59**	C	174.31	**V111**	HN	9.79	**V184**	HN	8.52	**K239**	CB	33.99
**N59**	CA	53.07	**V111**	N	123.74	**V184**	N	117.06	**K239**	HN	10.85
**N59**	CB	39.49	**T112**	C	175.84	**P185**	CA	63.18	**K239**	N	133.26
**N59**	HN	8.77	**T112**	CA	61.39	**K186**	CA	56.18	**K240**	C	178.29
**N59**	N	118.47	**T112**	CB	67.96	**K186**	CB	31.30	**K240**	CA	59.16
**L60**	C	176.60	**T112**	HN	8.58	**K186**	HN	7.85	**K240**	CB	31.32
**L60**	CA	55.00	**T112**	N	116.83	**K186**	N	118.17	**K240**	HN	8.73
**L60**	CB	44.52	**L113**	C	174.01	**T187**	CA	58.91	**K240**	N	121.05
**L60**	HN	8.29	**L113**	CA	55.80	**T187**	CB	71.51	**D241**	C	176.80
**L60**	N	113.49	**L113**	CB	44.27	**T187**	HN	7.59	**D241**	CA	55.10
**K61**	C	171.72	**L113**	HN	7.58	**T187**	N	115.01	**D241**	CB	39.48
**K61**	CA	52.16	**L113**	N	124.23	**P188**	C	172.83	**D241**	HN	8.24
**K61**	CB	33.96	**L114**	C	174.94	**P188**	CA	58.68	**D241**	N	113.03
**K61**	HN	9.19	**L114**	CA	53.49	**P188**	CB	34.79	**H242**	C	175.70
**K61**	N	120.42	**L114**	CB	44.04	**T189**	C	173.04	**H242**	CA	55.77
**P62**	C	175.83	**L114**	HN	7.61	**T189**	CA	61.15	**H242**	CB	33.46
**P62**	CA	63.17	**L114**	N	118.40	**T189**	CB	70.73	**H242**	HN	7.84
**P62**	CB	30.58	**T115**	CA	58.50	**T189**	HN	8.96	**H242**	N	116.81
**G63**	C	175.02	**T115**	CB	70.96	**T189**	N	124.47	**T243**	C	175.28
**G63**	CA	44.03	**T115**	HN	8.91	**D190**	CA	53.00	**T243**	CA	58.61
**G63**	HN	9.27	**T115**	N	109.96	**D190**	CB	45.72	**T243**	CB	71.88
**G63**	N	113.03	**V116**	CA	61.22	**D190**	HN	9.39	**T243**	HN	7.73
G63	*C*	*175.07*	**V116**	CB	34.21	**D190**	N	129.44	**T243**	N	105.78
G63	*CA*	*44.06*	**V116**	HN	8.34	**D192**	C	177.97	**D244**	C	179.25
G63	*HN*	*9.03*	**V116**	N	119.55	**D192**	CA	54.48	**D244**	CA	51.88

G63	*N*	*112.70*	**N127**	C	173.64	**D192**	CB	40.26	**D244**	CB	41.33
**D64**	C	175.23	**N127**	CA	52.16	**Q193**	C	174.61	**D244**	HN	8.58
**D64**	CA	55.70	**N127**	CB	38.73	**Q193**	CA	56.91	**D244**	N	120.38
**D64**	CB	41.16	**I128**	C	175.64	**Q193**	CB	28.76	**K245**	CA	58.36
**D64**	HN	8.53	**I128**	CA	61.64	**Q193**	HN	8.62	**K245**	CB	30.59
**D64**	N	125.28	**I128**	CB	36.33	**Q193**	N	123.77	**K245**	HN	8.55
**K65**	C	174.15	**I128**	HN	8.74	**V194**	C	174.94	**K245**	N	117.27
**K65**	CA	54.52	**I128**	N	125.02	**V194**	CA	61.08	**G247**	C	173.01
**K65**	CB	35.75	**I129**	C	175.50	**V194**	CB	33.19	**G247**	CA	45.11
**K65**	HN	8.53	**I129**	CA	63.14	**V194**	HN	8.43	**G247**	HN	8.26
**K65**	N	117.69	**I129**	CB	37.82	**V194**	N	122.79	**G247**	N	111.13
**L66**	C	174.95	**I129**	HN	8.82	**Q195**	C	174.01	**Y248**	C	178.00
**L66**	CA	52.91	**I129**	N	129.01	**Q195**	CA	54.27	**Y248**	CA	58.09
**L66**	CB	44.02	**L130**	C	179.25	**Q195**	CB	32.42	**Y248**	CB	37.88
**L66**	HN	9.54	**L130**	CA	52.80	**Q195**	HN	9.46	**Y248**	HN	8.25
**L66**	N	122.79	**L130**	CB	41.15	**Q195**	N	127.37	**Y248**	N	123.23
**T67**	C	175.18	**L130**	HN	7.64	**M196**	C	174.46	**V249**	C	176.07
**T67**	CA	60.83	**L130**	N	115.41	**M196**	CA	53.29	**V249**	CA	61.55
**T67**	CB	70.60	**D131**	C	173.03	**M196**	CB	35.29	**V249**	CB	27.79
**T67**	HN	9.00	**D131**	CA	53.18	**M196**	HN	9.37	**V249**	HN	8.42
**T67**	N	115.62	**D131**	CB	43.80	**M196**	N	122.21	**V249**	N	129.44
**K68**	C	173.49	**D131**	HN	8.31	**E197**	C	175.89	**K250**	C	179.75
**K68**	CA	53.76	**D131**	N	119.49	**E197**	CA	54.44	**K250**	CA	56.91
**K68**	CB	34.22	**D132**	C	175.50	**E197**	CB	30.07	**K250**	CB	30.98
**K68**	HN	9.44	**D132**	CA	55.59	**E197**	HN	9.35	**K250**	HN	8.42
**K68**	N	127.23	**D132**	CB	39.98	**E197**	N	125.30	**K250**	N	135.93
K68	*CA*	*53.60*	**D132**	HN	7.50	**I198**	C	173.60	**E251**	C	177.60
K68	*CB*	*34.48*	**D132**	N	118.38	**I198**	CA	59.39	**E251**	CA	57.97
K68	*HN*	*9.30*	**A133**	C	176.23	**I198**	CB	38.98	**E251**	CB	28.92
K68	*N*	*126.95*	**A133**	CA	49.73	**I198**	HN	9.47	**E251**	HN	8.20
**D69**	C	174.44	**A133**	CB	18.69	**I198**	N	127.35	**E251**	N	120.00
**D69**	CA	52.77	**A133**	HN	8.12	**Q199**	C	174.56	**N252**	C	178.86
**D69**	CB	44.40	**A133**	N	124.59	**Q199**	CA	52.71	**N252**	CA	52.50
**D69**	HN	8.12	**N134**	C	177.15	**Q199**	CB	31.79	**N252**	CB	37.29
**D69**	N	121.83	**N134**	CA	55.07	**Q199**	HN	9.28	**N252**	HN	7.12
**F70**	C	173.62	**N134**	CB	41.20	**Q199**	N	126.16	**N252**	N	110.75
**F70**	CA	55.80	**N134**	HN	9.74	**F200**	C	175.22	**K254**	C	175.19
**F70**	CB	43.00	**N134**	N	125.45	**F200**	CA	52.45	**K254**	CA	54.75
**F70**	HN	9.34	**L135**	C	178.53	**F200**	CB	36.44	**K254**	CB	34.15
**F70**	N	119.58	**L135**	CA	58.05	**F200**	HN	8.86	**A255**	C	176.03
**Q71**	C	174.08	**L135**	CB	40.23	**F200**	N	128.57	**A255**	CA	51.23
**Q71**	CA	53.14	**L135**	HN	9.05	**K201**	C	174.76	**A255**	CB	18.06
**Q71**	CB	31.53	**L135**	N	125.77	**K201**	CA	56.65	**A255**	HN	8.04
**Q71**	HN	8.64	**K136**	C	178.07	**K201**	CB	31.38	**A255**	N	120.23
**Q71**	N	120.19	**K136**	CA	59.63	**K201**	HN	9.46	***A255***	*C*	*177.22*
**F72**	C	175.01	**K136**	CB	28.84	**K201**	N	131.47	***A255***	*CA*	*51.21*
**F72**	CA	55.87	**K136**	HN	8.15	***K201***	*C*	*174.64*	***A255***	*CB*	*19.79*
**F72**	CB	41.16	**K136**	N	118.68	***K201***	*CA*	*56.48*	**H256**	C	172.52
**E73**	C	173.66	**D137**	C	177.97	***K201***	*CB*	*31.56*	**H256**	CA	58.62
**E73**	CA	53.54	**D137**	CA	57.40	***K201***	*HN*	*9.38*	**H256**	CB	23.98
**E73**	CB	33.40	**D137**	CB	39.43	***K201***	*N*	*130.64*	**H256**	HN	3.18

**E73**	HN	9.03	**D137**	HN	7.15	**D202**	C	175.20	**H256**	N	105.23
**E73**	N	119.85	**D137**	N	118.68	**D202**	CA	52.61	***H256***	*C*	*173.22*
**N74**	C	175.00	**L138**	C	180.06	**D202**	CB	39.75	***H256***	*CA*	*57.76*
**N74**	CA	52.72	**L138**	CA	57.68	**D202**	HN	8.66	***H256***	*CB*	*25.04*
**N74**	CB	38.77	**L138**	CB	41.02	**D202**	N	121.88	***H256***	*HN*	*3.14*
**N74**	HN	9.54	**L138**	HN	8.12	**D203**	C	177.18	***H256***	*N*	*104.07*
**N74**	N	120.61	**L138**	N	119.37	**D203**	CA	52.24	**S257**	C	171.17
**N75**	C	175.42	**Y139**	C	175.41	**D203**	CB	41.15	**S257**	CA	54.13
**N75**	CA	51.83	**Y139**	CA	59.37	**D203**	HN	7.65	**S257**	CB	59.77
**N75**	CB	39.69	**Y139**	CB	36.81	**D203**	N	124.75	**S257**	HN	6.33
**N75**	HN	8.80	**Y139**	HN	8.43	**K204**	C	175.86	**S257**	N	111.94
**N75**	N	125.69	**Y139**	N	123.97	**K204**	CA	55.51	**E258**	C	175.79
**G76**	C	172.14	**L140**	C	180.27	**K204**	CB	30.63	**E258**	CA	51.89
**G76**	CA	44.03	**L140**	CA	57.40	**K204**	HN	8.95	**E258**	CB	33.25
**G76**	HN	8.52	**L140**	CB	40.08	**K204**	N	127.46	**E258**	HN	7.58
**G76**	N	110.59	**L140**	HN	8.65	**T205**	C	174.68	**E258**	N	111.57
**S77**	C	173.11	**L140**	N	120.90	**T205**	CA	65.02	***E258***	*C*	*175.72*
**S77**	CA	58.88	**M141**	C	176.89	**T205**	CB	67.90	***E258***	*CA*	*52.09*
**S77**	CB	64.16	**M141**	CA	59.08	**T205**	HN	8.21	***E258***	*CB*	*33.40*
**S77**	HN	8.47	**M141**	CB	33.03	**T205**	N	117.12	***E258***	*HN*	*7.75*
**S77**	N	115.32	**M141**	HN	8.59	**K206**	C	176.47	***E258***	*N*	*111.56*
**L78**	C	175.67	**M141**	N	117.05	**K206**	CA	54.51	**D259**	C	174.69
**L78**	CA	51.72	**S142**	C	175.42	**K206**	CB	36.23	**D259**	CA	54.85
**L78**	CB	43.34	**S142**	CA	60.71	**K206**	HN	8.88	**D259**	CB	39.41
**L78**	HN	7.91	**S142**	CB	64.45	**K206**	N	127.91	**D259**	HN	8.64
**L78**	N	121.42	**S142**	HN	8.04	**D207**	C	178.41	**D259**	N	122.72
**A79**	C	173.79	**S142**	N	111.75	**D207**	CA	51.64	**K260**	CA	59.95
**A79**	CA	52.99	**A143**	C	179.69	**D207**	CB	40.64	**K260**	CB	33.87
**A79**	CB	18.37	**A143**	CA	52.96	**D207**	HN	8.85	**K260**	HN	8.12
**A79**	HN	8.41	**A143**	CB	19.54	**D207**	N	121.00	**K260**	N	118.66
**A79**	N	122.49	**A143**	HN	9.08	**E208**	C	177.49			
**I80**	C	176.84	**A143**	N	124.93	**E208**	CA	58.24			
**I80**	CA	59.44	***A143***	*C*	*179.70*	**E208**	CB	27.78			

Table S3. X-Ray diffraction data collection and refinement statistics.

	$TapA_{71-190}$	$TapA_{75-190}$
**Data collection**		
Space group	$P2_12_12_1$	$P2_1$
Cell dimensions		
a, *b*, *c* (Å)	39.2, 52.9, 167.7	30.1, 61.1, 34.3
α, β, γ (°)	90.0, 90.0, 90.0	90.0, 106.9, 90.0
Resolution (Å)*	44.77-1.07 (1.13-1.07)	32.8-1.28 (1.36-1.28)
R_{meas}*	6.6 (154.2)	4.5 (177.0)
$< I / \sigma(I) >$*	9.77 (0.8)	14.2 (0.8)
Completeness (%)*	98.8 (97.9)	98.4 (97.2)
Multiplicity*	3.5	4.4
**Refinement**		
No. reflections	151,026	30,250
R_{work}/ R_{free}	0.148/0.176	0.147/0.197
No. atoms		
Protein	2949	943
Ligand/ion	28	15
Water	742	212
Mean B factor ($Å^2$)	17.8	28.0
R.m.s deviations		
Bond lengths (Å)	0.012	0.012
Bond angles (º)	1.632	1.685
Ramachandran		
Outlier (%)	0	0
Favored (%)	96.6	95.6

* Data in highest resolution shell are indicated in parenthesis.

Table S4. H^N-H^N contacts observed by NMR within TasA filaments including those shown in SI Appendix, Fig. S8. Contacts shown in column 1 are located in antiparallel β-sheets and are involved in cross-strand hydrogen bonds, the remaining show validated contacts in loop and helical regions.

D31	-	A230	K65	-	L236	N145	-	D146	G219	-	N220
K33	-	F228	K65	-	I138	A147	-	A148	H242	-	T243
K35	-	F226	G42	-	N59	A148	-	A149	K250	-	E251
A37	-	L224	G42	-	L60	A149	-	A150	E251	-	N252
F39	-	I222	N59	-	L60	A150	-	E151			
S41	-	N220	E103	-	D104	E151	-	K152			
S58	-	V249	D104	-	F105	K152	-	I153			
K49	-	Q71	F105	-	L106	I153	-	K154			
G63	-	F200	S107	-	Q108	K154	-	K155			
K68	-	M196	Q108	-	F109	K155	-	Q156			
F70	-	V194	Y139	-	L140	Q156	-	I157			
M85	-	V168	L140	-	M141	E180	-	Y181			
L84	-	E229	M141	-	S142	Y181	-	D182			
G90	-	K223	S142	-	A143	N215	-	K216			
K93	-	S221	A143	-	K144	K216	-	Y217			
V111	-	A133	K144	-	N145	Y217	-	Q218			

Table S5. Primer used for cloning different TapA fragments into pCA528.

**TapA44-253**	**BsmI XhoI TapA44-253**
**Forward 1**	5‘CCAGTGCGTCTCAGGTGGTGCTTTTCATGATATTGAAAC
**Reverse 1**	5‘TCGACTCGAGttaCTGATCAGCTTCATTGC
**TapA44-190/33-190**	mutation for stop codon generation at AA 191
**Forward 2**	5‘CAAAATGCGATGAATAACCGACAGTCCCTAAAAAAG
**Reverse 2**	5‘AGGGACTGTCGGTTATTCATCGCATTTTGCAAGCCTC
**TapA54-190**	TapA54ff C-terminus Sumo
**Forward 3**	5‘TCACTTCAAACGTGTAAAGACTTTCAGCATACAG
**Reverse 3**	5‘TACACGTTTGAAGTGAACCACCAATCTGTTCTCTGTGAGCCTC
**TapA60-190**	TapA60ff C-terminus Sumo
**Forward 4**	5‘GACTTTCAGCATACAGATAAAAACTGCCATTATG
**Reverse 4**	5‘TCTGTATGCTGAAAGTCACCACCAATCTGTTCTCTGTGAGCCTC
**TapA66-190**	TapA66ff C-terminus Sumo
**Forward 5**	5‘AAAAACTGCCATTATGATAAACGCTGGGATCAAAG
**Reverse 5**	5‘TCATAATGGCAGTTTTTACCACCAATCTGTTCTCTGTGAGCCTC
**TapA71-190**	TapA71ff C-terminus Sumo
**Forward 6**	5‘GATAAACGCTGGGATCAAAGTGATTTGCACATATCAGATC
**Reverse 6**	5‘CCAGCGTTTATCACCACCAATCTGTTCTCTGTGAGCCTC
**TapA75-190**	C-terminus Sumo TapA75ff
**Forward 7**	5‘AGAGAACAGATTGGTGGTGCTGATCAAAGTGATTTGCACATATCAGATCAAACGG
**Reverse 7**	5‘TGTGCAAATCACTTTGATCAGCACCACCAATCTGTTCTCTGTGAGCCTCAATAATATCG

Table S6. Primers for Biofilm experiments.

Primer	Sequence	Used for
**RK221**	ACAT gcatgc TTATTCATCGCATTTTGCAAGCCTC	pKXD2, pKXD3, pKXD4, pKXD5
**RK222**	ACAT gcatgc TTACTGATCAGCTTCATTGC	pKXD6, pKXD7
**RK224**	CCGATGATACAAGCGCTGCT GATCAAAGTGATTTG	pKXD3
**RK225**	GATATGTGCAAATCACTTTGATC AGCAGCGCTTGTATC	pKXD3
**RK226**	CCGATGATACAAGCGCTGCT GATAAACGCTGGGATC	pKXD4
**RK227**	CACTTTGATCCCAGCGTTTATC AGCAGCGCTTGTATC	pKXD4
**RK228**	CATTTTCAGTCTGACTGCCGCAATA GCTTTTCATGATATTG	pKXD5, pKXD6
**RK229**	CAAATGTTTCAATATCATGAAAAGC TATTGCGGCAGTCAG	pKXD5, pKXD6
**RK234**	CACAATCAGCAAAAGGCGAAGCAATCAGACCAGAAGG	pKXD13
**RK235**	CCCGCTTTCCTTCTGGTCTGATTGCTTCGCCTTTTGCTGATTG	pKXD13
**RK239**	TGGCGAATTCTCAGAGTTAAATGGTATTGCTTCACT	pKXD13
**RK240**	GACTAGTCC TTAATTTTTATCCTCGCTATGCGC	pKXD13
**RK242**	TCCCCCCGGGGGGATCAGAGTTAAATGGTATTGCTTCACT	pKXD2-7
**RK243**	GCTCTAGAGCCTCAGAGTTAAATGGTATTGCTTCACT	pKXD13
**KD117**	aCGTTTGAAGTGAGACATC	pKXD23
**KD118**	AAGCATGCAAGCTAATTC	pKXD23

Table S7. Plasmids for Biofilm experiments.

Plasmid	Genotype	Construction of strain
**pKXD2**	pBS2E-P_{tapA}-*tapA190*	BKD34
**pKXD3**	pBS2E-P_{tapA}-*tapA190Δ45-74*	BKD35
**pKXD4**	pBS2E-P_{tapA}-*tapA190Δ45-70*	BKD36
**pKXD5**	pBS2E-P_{tapA}-*tapA190Δ34-43*	BKD37
**pKXD6**	pBS2E-P_{tapA}-*tapA253Δ34-43*	BKD38
**pKXD7**	pBS2E-P_{tapA}-*tapA*	BKD39
**pKXD13**	pBS1K-P_{tapA}-*tapAΔ13-234-sipW-tasA*	BKD30
**pKXD23**	pBS2E-P_{tapA}-*tapA1-57*	BKD121

Table S8. Strains for Biofilm experiments.

Strain	Genotype	Reference
**DK1042**	NCIB 3610 *comIQ12L*	(4)
**BRK49**	*comIQ12L ΔtapA-sipW-tasA::spc*	(5)
**BRK263**	*ΔsipW::erm*	(5)
**BKD30**	*comIQ12L ΔtapA-sipW-tasA::spc*<br>*amyE::* P_{tapA}*-tapAΔ13-234-sipW-tasA-kan*	This study
**BKD34**	*comIQ12L ΔtapA-sipW-tasA::spc*<br>*amyE::* P_{tapA}*-tapAΔ13-234-sipW-tasA-kan*<br>*lacA::* P_{tapA}*-tapA190-erm*	This study
**BKD35**	*comIQ12L ΔtapA-sipW-tasA::spc*<br>*amyE::* P_{tapA}*-tapAΔ13-234-sipW-tasA-kan*<br>*lacA::*P_{tapA}*-tapA190Δ45-74-erm*	This study
**BKD36**	*comIQ12L ΔtapA-sipW-tasA::spc*<br>*amyE::* P_{tapA}*-tapAΔ13-234-sipW-tasA-kan*<br>*lacA::* P_{tapA}*-tapA190Δ45-70-erm*	This study
**BKD37**	*comIQ12L ΔtapA-sipW-tasA::spc*<br>*amyE::* P_{tapA}*-tapAΔ13-234-sipW-tasA-kan*<br>*lacA::* P_{tapA}*-tapA190Δ34-43-erm*	This study
**BKD38**	*comIQ12L ΔtapA-sipW-tasA::spc*<br>*amyE::* P_{tapA}*-tapAΔ13-234-sipW-tasA-kan*<br>*lacA::* P_{tapA}*-tapAΔ34-43-erm*	This study
**BKD39**	*comIQ12L ΔtapA-sipW-tasA::spc*<br>*amyE::* P_{tapA}*-tapAΔ13-234-sipW-tasA-kan*<br>*lacA::* P_{tapA}*-tapA-erm*	This study
**BKD121**	*comIQ12L ΔtapA-sipW-tasA::spc*<br>*amyE::* P_{tapA}*-tapAΔ13-234-sipW-tasA-kan*<br>*lacA::* P_{tapA}*-tapA57-erm*	This study

SI References

1. C. Gille, M. Fähling, B. Weyand, T. Wieland, A. Gille, Alignment-Annotator web server: rendering and annotating sequence alignments. *Nucleic Acids Res.* **42**, W3–W6 (2014).

2. A. E. Bennett, R. G. Griffin, J. H. Ok, S. Vega, Chemical shift correlation spectroscopy in rotating solids: Radio frequency-driven dipolar recoupling and longitudinal exchange. *J. Chem. Phys.* **96**, 8624–8627 (1992).

3. A. S. Maltsev, J. Ying, A. Bax, Deuterium isotope shifts for backbone 1H, 15N and 13C nuclei in intrinsically disordered protein α-synuclein. *J. Biomol. NMR* **54**, 181–191 (2012).

4. M. A. Konkol, K. M. Blair, D. B. Kearns, Plasmid-Encoded ComI Inhibits Competence in the Ancestral 3610 Strain of Bacillus subtilis. *J. Bacteriol.* **195**, 4085–4093 (2013).

5. R. M. Alver, "The role of ClpC and its adaptor proteins in protein quality control systems of Bacillus subtilis," Hannover : Leibniz Universität Hannover. (2020) https:/doi.org/10.15488/10150 (July 18, 2022).
